# Twisted Raman: Orbital Angular Momentum Control of Raman Optical Activity

**DOI:** 10.1021/acs.jpclett.6c02215

**Published:** 2026-09-06

**Authors:** Kayn A. Forbes

**Affiliations:** School of Chemistry, 6106 University of East Anglia, Norwich Research Park, Norwich NR4 7TJ, United Kingdom

## Abstract

Raman optical activity (ROA) conventionally detects differences in Raman scattering associated with opposite circular-polarization states σ, providing a sensitive probe of molecular chirality. Here we show that a different handedness of light can drive ROA: the wavefront handedness encoded in the topological charge *l* of a focused optical vortex. Instead of using photons carrying spin angular momentum σℏ, the response is controlled by photons carrying orbital angular momentum *l*ℏ, with the sign of *l* defining the optical handedness. We develop a nonparaxial theory of ROA with Laguerre–Gaussian beams and show that the Maxwell-consistent focused field allows a linearly polarized vortex beam to produce a topological-charge-dependent ROA signal from an isotropic ensemble of independent, randomly oriented chiral molecules, as in a gas or dilute solution. The response contains both electric-dipole–magnetic-dipole and electric-dipole–electric-quadrupole interference terms, establishing optical orbital angular momentum as a control parameter for chiral Raman spectroscopy.

## Graphical abstract

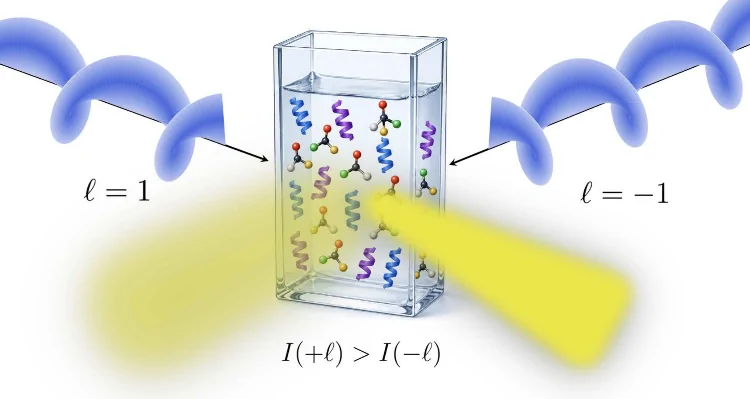

Chirality is a central structural property of molecules and materials, with consequences across stereochemistry, chemical recognition, photochemistry, biomolecular structure, and pharmaceutical function. A chiral system is not superposable on its mirror image, and this broken mirror symmetry allows left- and right-handed optical fields to interact differently with opposite molecular enantiomers.^1,2^ Chiroptical spectroscopies exploit this principle by detecting differential optical responses that depend on molecular handedness.^3–5^ Among these methods, Raman optical activity (ROA) is especially powerful because it combines the chemical specificity of vibrational Raman scattering with sensitivity to molecular chirality. ROA measures a small difference in Raman intensity associated with opposite circular-polarization states of the incident and/or scattered light, and therefore provides vibrationally resolved information on absolute configuration, conformation, solvation, supramolecular organization, and biomolecular structure.^6^

Recent developments have renewed interest in ROA as both a molecular spectroscopy and a platform for enhanced chiral light-matter interactions. Circular-polarization-resolved ROA has been highlighted as a route to information-rich chiral vibrational spectroscopy of molecules, crystals, and nanostructured materials.^7^ At the same time, resonance ROA and surface-enhanced ROA have been pursued to overcome the intrinsically weak magnitude of conventional ROA signals, with recent studies emphasizing signal amplification, chiral plasmonic substrates, and chirality transfer in complex molecular and nanophotonic systems.^8–16^ These advances show that ROA is no longer limited to conventional molecular solutions, but is increasingly connected to nanophotonic platforms and engineered optical fields. Nevertheless, the standard theoretical description remains anchored in plane-wave or Gaussian beam illumination, where optical activity is usually controlled by the pseudoscalar helicity σ of circular polarization.

Structured light^17^ offers a different route to chiral optical control.^18,19^ Instead of using only the spin angular momentum associated with circular polarization, structured beams allow the phase, amplitude, and polarization distributions of light to be engineered. Optical vortex beams are a particularly important example.^20,21^ Optical vortex beams carry an azimuthal phase factor exp­(i*l*ϕ), where the integer topological charge *l* determines the handedness and magnitude of the helical wavefront and endows each photon with orbital angular momentum *l*ℏ in the direction of propagation. The sign of *l* is a pseudoscalar degree of freedom: reversing *l* reverses the handedness of the optical vortex, independent of the state of polarization (Figure 1). It has been shown that the chirality associated with *l* can lead to new chiroptical techniques, enhanced chiroptical signals, and access to otherwise hidden structural information.^19^ Vortex beams are therefore natural candidates for chiroptical spectroscopy beyond conventional circular dichroism and circular ROA.

**Figure 1:**
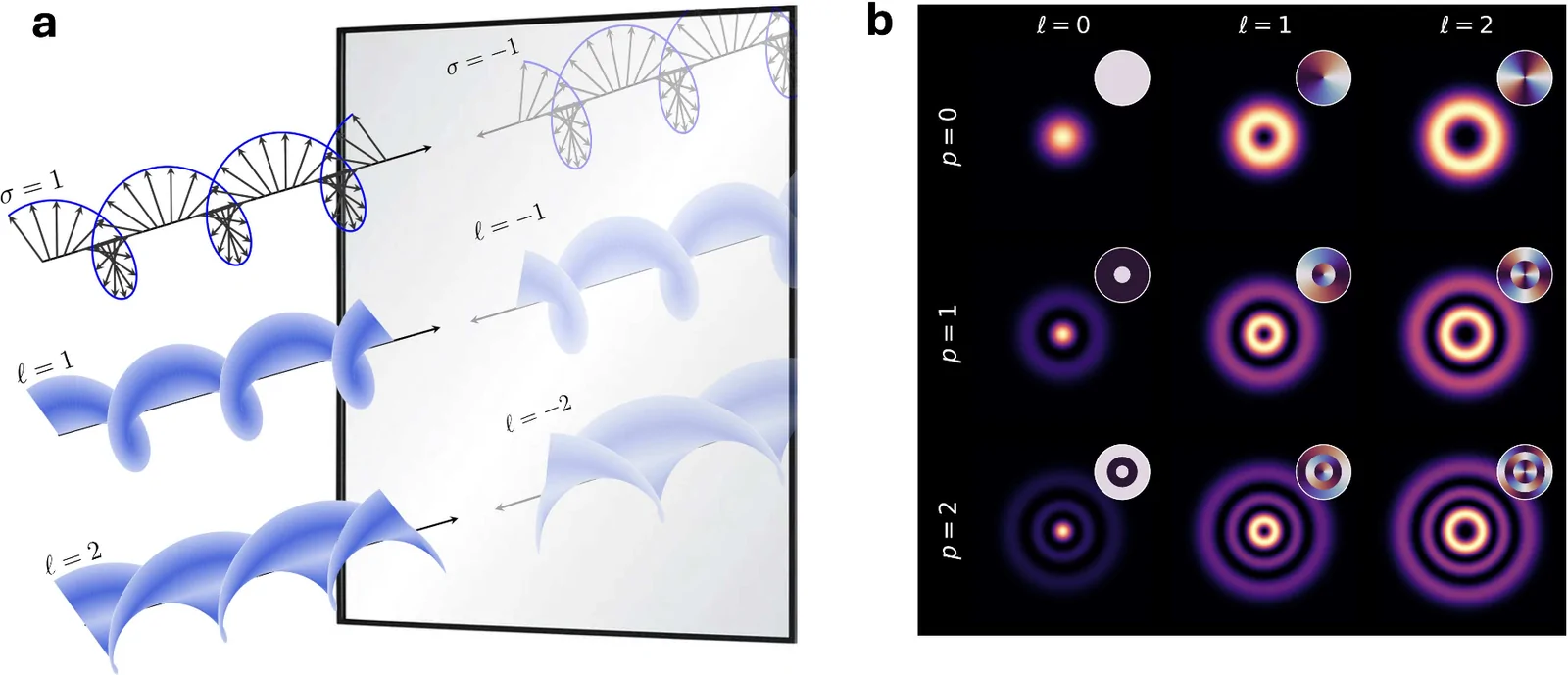
Handedness and spatial structure of Laguerre–Gaussian modes. (a) Independent handedness associated with circular polarization σ and the helicoidal wavefront of an optical vortex. The latter arises from the azimuthal factor exp­(i*l*ϕ) in the scalar spatial mode ψ_*lp*_, eq 2, with reflection reversing the corresponding signs, σ → −σ and *l* → −*l*. (b) Normalized waist-plane intensities |ψ_*lp*_|^2^ for the indicated combinations of *l* and *p*; insets show arg­(ψ_*lp*_). The radial mode function *f*_LG_^|*l*|*p*^ in eq 3 determines the central vortex and *p* radial nodes, while exp­(i*l*ϕ) determines the azimuthal phase winding. Each intensity is normalized independently.

The chiral electromagnetic structure of vortex beams is especially rich outside the simplest paraxial limit. Tight focusing, longitudinal fields, radial structure, spin-orbit coupling, and the sign of the topological charge can all reshape local optical chirality and spin densities.^18,19,22,23^ These effects are absent, or strongly suppressed, in ideal plane waves. They suggest that the chiral response of matter can be controlled not only through the handedness of polarization σ, but also through the topology *l* and spatial gradients of the optical field. This distinction is crucial for Raman scattering, where the molecular response contains not only electric-dipole–electric-dipole terms but also the electric-dipole–magnetic-dipole and electric-dipole–electric-quadrupole interference terms that generate ROA.

A previous theory specifically of Raman optical activity showed that optical vortex beams can generate a topological-charge-dependent ROA signal in chiral molecules.^24^ This ROA effect was also considered within a broader treatment of OAM-dependent optical activity.^25,26^ The earlier ROA theory established that the handedness of a molecular Raman signal need not be controlled solely by the spin angular momentum of circularly polarized light, but can also depend on the orbital handedness of the optical wavefront. However, the treatment was confined to the paraxial, effectively transverse regime. In that limit, the vortex dependence enters through transverse field gradients in the electric-dipole–electric-quadrupole interaction, and the resulting signal is proportional to σ*l*. It therefore still requires circular polarization and does not provide a purely topological ROA mechanism; moreover, the electric-dipole–magnetic-dipole channel remains hidden.

Here, we show that this is not the full story. In the nonparaxial regime relevant to focused vortex beams, the higher-order longitudinal and transverse field corrections generate a topological-charge-dependent ROA signal even for linearly polarized input, which would give no conventional ROA response in a plane-wave description. The signal is odd in the vortex charge and contains both electric-dipole–magnetic-dipole and electric-dipole–electric-quadrupole interference terms, making optical topological charge a control parameter for molecular ROA distinct from polarization helicity.

This extends ROA beyond the conventional circular-polarization paradigm. The theory predicts detection-resolved vortex ROA signals that disappear for ordinary plane-wave or Gaussian illumination, providing a clear experimental signature of topology-enabled chiral Raman scattering. More broadly, it establishes focused optical vortex beams as a route to multipolar control in molecular chiroptical spectroscopies.

We describe the Raman process using the standard molecular quantum electrodynamics formulation, in which the electromagnetic field is quantized and the molecule is represented by its multipolar transition moments.^1,27^ For a molecule ξ located at **R**_ξ_, the interaction Hamiltonian, to the order required for Raman optical activity, may be written as

1 $$H_{\mathrm{int}}\left(\xi \right)=-\mu_{i}\left(\xi \right)E_{i}\left(R_{\xi }\right)-m_{i}\left(\xi \right)B_{i}\left(R_{\xi }\right)-Q_{ij}\left(\xi \right)\nabla_{j}E_{i}\left(R_{\xi }\right)+\cdot \cdot \cdot$$

where μ_*i*_, *m*_*i*_, and *Q*_*ij*_ are the molecular electric-dipole, magnetic-dipole, and electric-quadrupole operators, respectively. The first term gives the ordinary E1E1 Raman amplitude, while the E1M1 and E1E2 interference terms give the chiral Raman optical activity contribution.^1,2^ Here E1, M1, and E2 denote electric-dipole, magnetic-dipole, and electric-quadrupole light–matter interactions, respectively. Thus, E1E1 is the ordinary Raman amplitude with two electric-dipole interactions, whereas E1M1 and E1E2 contain one electric-dipole interaction and one magnetic-dipole or electric-quadrupole interaction. ROA is produced by interference of E1E1 with these mixed amplitudes. The incident radiation is taken to be a focused Laguerre–Gaussian (LG) mode propagating in the *z* direction, where (*r*, ϕ, *z*) are cylindrical coordinates. We write the scalar LG factor as

2 $$\psi_{lp}\left(r\right)=f_{\mathrm{LG}}^{\left|l\right|p}\left(r,z\right)\;\exp\left[i\left(kz+l\phi \right)\right]$$

where l∈Z is the topological charge and p∈N is the radial index. The radial mode function is

3 $$f_{\mathrm{LG}}^{\left|l\right|p}\left(r,z\right)=\sqrt{\frac{2p!}{\pi {w_{0}}^{2}\left(p+\left|l\right|\right)!}}\frac{w_{0}}{w\left(z\right)}{\left(\frac{\sqrt{2}r}{w\left(z\right)}\right)}^{\left|l\right|}L_{p}^{\left|l\right|}\left[\frac{2r^{2}}{w^{2}\left(z\right)}\right]\;\exp\left(-\frac{r^{2}}{w^{2}\left(z\right)}\right)\;\exp\{i\left[\frac{kr^{2}}{2R\left(z\right)}-\left(2p+\left|l\right|+1\right)\zeta \left(z\right)]\right\}$$

where *w*_0_ is the beam waist, *w*(*z*) is the beam radius, *R*(*z*) is the wavefront radius of curvature, *ζ*(*z*) is the Gouy phase, *L*_*p*_^|*l*|^ is a generalized Laguerre polynomial, and ψ_*lp*_ is a scalar spatial mode function, i.e., a solution to the paraxial wave equation in cylindrical coordinates, not the electromagnetic scalar potential. In Coulomb gauge for the source-free monochromatic mode, Φ = 0 and the positive-frequency vector potential has the same spatial dependence, with **A** ^(+)^ = **E** ^(+)^/(iω). The calculation is formulated directly in terms of the electric and magnetic field operators, retained consistently through *O*(ϵ^2^) in the paraxiality parameter defined below.^18,28^

It is useful to introduce the logarithmic radial derivative

4 $$\gamma_{\left|l\right|p}\left(r,z\right)=\frac{1}{f_{\mathrm{LG}}^{\left|l\right|p}}\frac{\partial f_{\mathrm{LG}}^{\left|l\right|p}}{\partial r}=\frac{\left|l\right|}{r}-\frac{2r}{w^{2}\left(z\right)}+\frac{ikr}{R\left(z\right)}-\frac{4r}{w^{2}\left(z\right)}\frac{L_{p-1}^{\left|l\right|+1}\left[2r^{2}/w^{2}\left(z\right)\right]}{L_{p}^{\left|l\right|}\left[2r^{2}/w^{2}\left(z\right)\right]}$$

with the final Laguerre polynomial term taken to vanish for *p* = 0. The transverse derivatives of the scalar LG mode are written in terms of two gradient factors,

5 $$Q_{lp}=\gamma_{\left|l\right|p}\;\cos\phi -\frac{il}{r}\;\sin\phi$$

6 $$P_{lp}=\gamma_{\left|l\right|p}\;\sin\phi +\frac{il}{r}\;\cos\phi$$

These follow from ∂_*x*_ = cos ϕ ∂_*r*_ – *r* ^–1^ sin ϕ ∂_ϕ_ and ∂_*y*_ = sin ϕ ∂_*r*_ + *r* ^–1^ cos ϕ ∂_ϕ_ together with the azimuthal phase factor exp­(i*l*ϕ). Hence

7 $$\nabla_{⊥}\psi_{lp}=\left(Q_{lp}\mathrm{x̂}+P_{lp}ŷ\right)\psi_{lp}$$

The longitudinal derivative is written as

8 $$\partial_{z}\psi_{lp}=\left(ik+\delta Z_{lp}\right)\psi_{lp},,\delta Z_{lp}=O\left(k{\text{ϵ}}^{2}\right)$$

where ϵ = (*kw*_0_)^−1^ ≪ 1 is the dimensionless paraxiality parameter.^28^ Physically, ϵ measures the strength of focusing: ϵ → 0 is the paraxial limit, whereas increasing ϵ corresponds to a tighter beam waist and larger longitudinal and higher-order transverse field components. The notation *O*(ϵ^*n*^) denotes a contribution that scales as ϵ^*n*^ relative to the leading transverse field. We therefore define the leading scalar-mode gradient factor

9 $$T=Q_{lp}\mathrm{x̂}+P_{lp}ŷ+ikẑ$$

with the *O*(ϵ^2^) longitudinal-envelope correction retained in the complete field-gradient tensor introduced below.

The incident transverse Jones state is

10 $$t=\alpha \mathrm{x̂}+\beta ŷ,,u=ẑ\times t=-\beta \mathrm{x̂}+\alpha ŷ,,{\left|\alpha \right|}^{2}+{\left|\beta \right|}^{2}=1$$

Retaining the Maxwell-consistent field through *O*(ϵ^2^), the electric and magnetic operators are written compactly as

11 $$E_{\mathrm{LG}}\left(r\right)=\underset{k,l,p}{\sum }\Omega \left[e_{lp}\left(r\right)\psi_{lp}\left(r\right)a_{lp}-\mathrm{H.c.}\right]$$

12 $$B_{\mathrm{LG}}\left(r\right)=\underset{k,l,p}{\sum }\frac{\Omega }{c}\left[b_{lp}\left(r\right)\psi_{lp}\left(r\right)a_{lp}-\mathrm{H.c.}\right]$$

where *a*_*lp*_ is the photon annihilation operator and H.c. denotes the Hermitian conjugate. The full dimensionless local field factors in eqs 11 and 12 are given by

13 $$\begin{array}{c} e_{lp}=t+\frac{i}{k}S_{lp}ẑ+e_{lp}^{T_{2}},\\ b_{lp}=u+\frac{i}{k}R_{lp}ẑ+b_{lp}^{T_{2}}, \end{array}$$

where the second-order transverse amplitudes are defined by

14 $$\psi_{lp}e_{lp}^{T_{2}}=T_{2}\left[t\right]\psi_{lp},,\psi_{lp}b_{lp}^{T_{2}}=T_{2}\left[u\right]\psi_{lp}$$

The operator $T_{2}$ , including its scalar-envelope and vectorial parts, is given explicitly in the Supporting Information. The longitudinal factors are

15 $$S_{lp}=\alpha Q_{lp}+\beta P_{lp},,R_{lp}=\alpha P_{lp}-\beta Q_{lp}$$

The normalization factor is

16 $$\Omega =i{\left(\frac{\hbar ck}{2\varepsilon_{0}V{A_{\left|l\right|,p}}^{2}}\right)}^{1/2}$$

where *V* is the quantization volume and *A*_|*l*|,*p*_ fixes the mode normalization.

The electric-quadrupole interaction requires the gradient of the complete incident electric field. We therefore define

$$D_{il}\psi_{lp}\equiv \nabla_{l}\left(e_{lp,i}\psi_{lp}\right)=\left[t_{i}T_{l}+t_{i}{ẑ}_{l}\delta Z_{lp}+\frac{i}{k}{ẑ}_{i}L_{l}+D_{il}^{T_{2}}\right]\psi_{lp}$$ 17

where the auxiliary quantities L_1_ and D_il_^T_2_^ are defined by

18 $$\nabla_{l}\left(S_{lp}\psi_{lp}\right)=L_{l}\psi_{lp},,D_{il}^{T_{2}}\psi_{lp}=\nabla_{l}\left(e_{lp,i}^{T_{2}}\psi_{lp}\right)$$

The explicit field-gradient components are given in the Supporting Information. The longitudinal-envelope correction δ*Z*_*lp*_ is retained at *O*(ϵ^2^), although it is even under *l* → −*l* and therefore does not contribute to the topological-charge differential derived below.

The hierarchy of field corrections follows directly from the source-free Maxwell equations. Schematically, the electric field may be written as

19 E=E⊥(0)︸T0+Ez(1)z^︸L1+E⊥(2)︸T2+···

Gauss’s law, ∇·**E** = 0, then gives to leading nonparaxial order

20 $$E_{z}^{\left(1\right)}=\frac{i}{k}\nabla_{⊥}\cdot E_{⊥}^{\left(0\right)}$$

so transverse spatial structure necessarily generates the longitudinal L_1_ field (Figure 2). At the next order, the coupled source-free Maxwell equations generate the transverse T_2_ correction. Thus, T_0_, L_1_, and T_2_ are successive components of the same Maxwell-consistent field rather than independent additions.^18,28,29^ The same hierarchy applies to the magnetic field.

**Figure 2:**
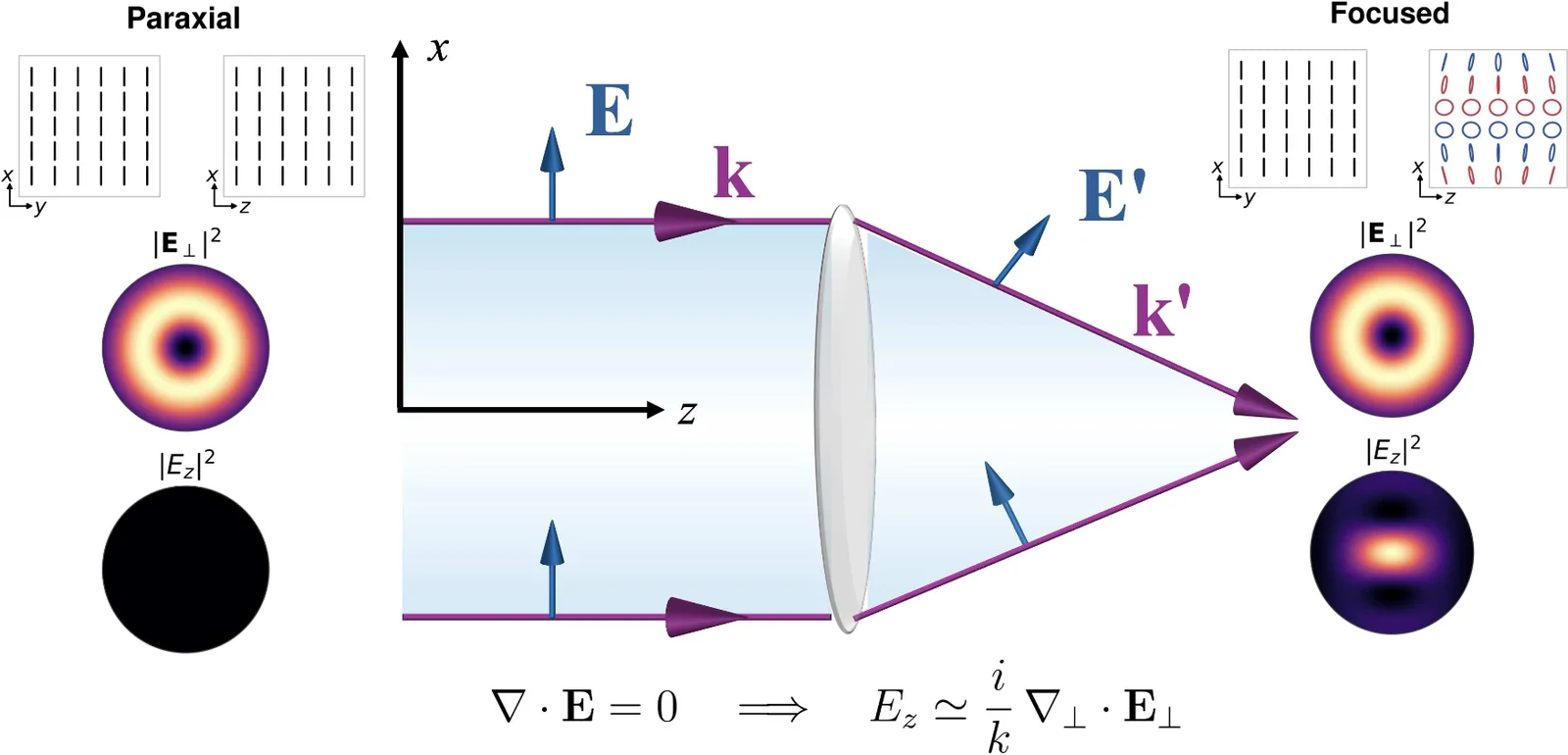
Generation of a longitudinal electric-field component by focusing an *x*-polarized LG_0_^1^ vortex beam. In the paraxial limit (left), the field is transverse to the propagation direction, **E** ≈ **E**_⊥_ = **x̂** *E*_*x*_, and therefore appears linearly polarized in both the *xy* and *xz* projections. The transverse intensity |**E**_⊥_|^2^ has the characteristic annular distribution of the *l* = 1 mode, whereas |*E*_*z*_|^2^ is negligible. Focusing redirects the constituent wavevectors from **k** to **k**′ and rotates their associated electric fields from **E** to **E**′. Enforcing the transversality condition, ∇·**E** = 0, gives the leading-order longitudinal component in eq 20. To leading nonparaxial order, the *xy* projection remains linearly polarized, whereas the relative amplitude and phase of *E*_*x*_ and *E*_*z*_ produce spatially varying elliptical polarization in the *xz* plane. Red and blue distinguish opposite local polarization handedness. The transverse and longitudinal component intensities are independently normalized and are shown schematically. An analogous longitudinal magnetic-field component follows from ∇·**B** = 0, such that $B_{z}\approx \frac{i}{k}\nabla_{⊥}\cdot B_{⊥}$ . The schematic illustrates the dominant geometrical consequence of focusing: the longitudinal field required by transversality. The smaller *O*(ϵ^2^) transverse correction is included in the theory but omitted from the schematic for clarity.

With this notation, the Maxwell-consistent field hierarchy is

21 $$T_{0}=O\left(1\right),,L_{1}=O\left(\text{ϵ}\right),,T_{2}=O\left({\text{ϵ}}^{2}\right)$$

The conventional paraxial terms and the nonparaxial contributions relevant to the present *l*-odd channel scale as follows:

22 $$\begin{array}{ll} \mathrm{E1M1:} & T_{0}T_{0}∼O\left(1\right),,L_{1}L_{1}∼T_{0}T_{2}∼O\left({\text{ϵ}}^{2}\right)\\ \mathrm{E1E2:} & T_{0}\partial_{z}T_{0}∼O\left(k\right),,k^{\prime }T_{0}T_{0}∼O\left(k^{\prime }\right),\\ \; & L_{1}\nabla_{⊥}T_{0}∼T_{2}\partial_{z}T_{0}∼T_{0}\partial_{z}T_{2}∼O\left(k{\text{ϵ}}^{2}\right),\\ \; & k^{\prime }T_{0}T_{2}∼O\left(k^{\prime }{\text{ϵ}}^{2}\right) \end{array}$$

The T_0_–T_0_ terms are the conventional paraxial ROA contributions and are generally leading. For the linearly polarized incident and detected fields used here, however, they are identically zero. They are also independent of the sign of *l* and therefore cannot contribute to the topological-charge differential.

The field model used here is therefore an analytic Maxwell-consistent focused LG beam model.^18^ It is not intended to represent the complete vectorial focal field produced by a particular high-numerical-aperture objective. In such cases, the detailed focal distribution depends on the optical system and is more naturally described by Richards–Wolf or angular-spectrum methods.^30,31^ The purpose of the present model is instead to isolate, within a controlled Lax expansion, the longitudinal and second-order transverse field terms and gradients that enter the E1M1 and E1E2 molecular contractions responsible for the vortex ROA.

We consider scattering from an initial molecular state |*E*_*i*_⟩ to a final molecular state |*E*_*f*_⟩. Rayleigh scattering is recovered when *E*_*f*_ = *E*_*i*_, whereas Raman scattering corresponds to *E*_*f*_ ≠ *E*_*i*_. The incident radiation mode is a Laguerre–Gaussian vortex mode specified by wave number *k*, polarization label η, topological charge *l*, and radial index *p*. The scattered photon is detected in a far-field plane-wave mode with wave vector **k**′ and polarization label η′. The initial and final states of the molecule–radiation system are therefore

23 $$\begin{array}{l} \left|i\right\rangle =\left|E_{i};n\left(k,\eta ,l,p\right)\right\rangle ,\\ \left|f\right\rangle =\left|E_{f};\left(n-1\right)\left(k,\eta ,l,p\right),1\left(k^{\prime },\eta^{\prime }\right)\right\rangle , \end{array}$$

where *n* is the occupation number of the incident vortex mode and the molecule is located at **r** = (*r*, ϕ, *z*). The incident electric and magnetic fields are represented by the complete local factors **e**_*lp*_ and **b**_*lp*_ in eq 13; the *lp* labels are suppressed inside the contractions below. The detected plane-wave factors are **e**′ and **b**′ = **k̂**′ × **e**′. In what follows, an overbar denotes complex conjugation, and repeated Cartesian indices are summed.

Using the multipolar light–matter Hamiltonian (eq 1), the E1E1 and E1M1 scattering amplitude is obtained from second-order perturbation theory as

24 $$M_{fi}^{\left(\alpha +G\right)}=-\Omega^{2}\sqrt{n}f_{\mathrm{LG}}^{\left|l\right|p}\left(r,z\right)\;\exp\left[i\left(kz+l\phi -k^{\prime }\cdot r\right)\right]\times \left[{\mathrm{e̅}}_{i}^{\prime }e_{j}\alpha_{ij}+\frac{1}{c}{\mathrm{e̅}}_{i}^{\prime }b_{j}G_{ij}+\frac{1}{c}{\mathrm{b̅}}_{i}^{\prime }e_{j}{\mathrm{G̅}}_{ji}\right]$$

where α_*ij*_ is the electric-dipole–electric-dipole Raman tensor and *G*_*ij*_ is the electric-dipole–magnetic-dipole optical activity tensor for the molecular transition |*E*_*i*_⟩ → |*E*_*f*_⟩. The constant Ω contains the single-photon field normalization; for Raman scattering with *k*′ ≠ *k*, Ω^2^ denotes the product of the incident and scattered field normalization factors. Since the density of final photon states ρ_*f*_ and other global constants are common to the observables compared below, we work at the level of the unaveraged squared matrix element for now; the rotational average is introduced explicitly below:

25 $${\left|M_{fi}^{\left(\alpha +G\right)}\right|}^{2}=n\Omega^{4}{\left|f_{\mathrm{LG}}^{\left|l\right|p}\left(r,z\right)\right|}^{2}\left[{\mathrm{e̅}}_{i}^{\prime }e_{j}\alpha_{ij}+\frac{1}{c}{\mathrm{e̅}}_{i}^{\prime }b_{j}G_{ij}+\frac{1}{c}{\mathrm{b̅}}_{i}^{\prime }e_{j}{\mathrm{G̅}}_{ji}\right]\times \left[e_{k}^{\prime }{\mathrm{e̅}}_{l}{\mathrm{\alpha ̅}}_{kl}+\frac{1}{c}e_{k}^{\prime }{\mathrm{b̅}}_{l}{\mathrm{G̅}}_{kl}+\frac{1}{c}b_{k}^{\prime }{\mathrm{e̅}}_{l}G_{lk}\right]$$

The terms responsible for optical activity are the interferences between the pure E1 scattering α-dependent terms and the mixed E1M1 scattering contributions dependent on *G*:

26 |Mfi|αG2=2nΩ4c|fLG|l|p(r,z)|2⁡Re[e̅i′ejek′b̅lαijG̅kl+e̅i′ejbk′e̅lαijGlk]

In order to account for an isotropic ensemble of freely tumbling chiral molecules, we require the rotational average of eq 26. The isotropically averaged results below apply to independent, randomly oriented molecules whose Raman signals add incoherently, as in a gas or dilute solution. Before rotational averaging, the amplitudes describe a local molecular emitter at a specified position and orientation. Using standard methods,^32–34^ the fourth-rank rotational average yields

27 ⟨|Mfi|2⟩αG=nΩ415c|fLG|l|p(r,z)|2Re[(e̅′·e)(e′·b̅)(4αλλG̅μμ−2αλμG̅λμ)+(e̅′·e)(b′·e̅)(4αλλGμμ−2αλμGλμ)+(e̅′·e′)(e·b̅)(−αλλG̅μμ+3αλμG̅λμ)+(e̅′·e̅)(e·b′)(−αλλGμμ+3αλμGλμ)+(e̅′·b̅)(e·e′)(−αλλG̅μμ+3αλμG̅λμ)+(e̅′·b′)(e·e̅)(−αλλGμμ+3αλμGλμ)]

This E1M1 result allows arbitrary incident polarization and is specialized below to far-field plane-wave detection. In the plane-wave limit with circularly polarized input it reduces to the familiar results of Craig and Thirunamachandran^1^ and Barron.^2^

It is well known^35–37^ that electric-quadrupole contributions to Raman optical activity cannot be neglected. Using the full gradient tensor *D*_*kl*_ defined in eq 17, the E1E1 and E1E2 amplitude is

28 $$M_{fi}^{\left(\alpha +A\right)}=-\Omega^{2}\sqrt{n}f_{\mathrm{LG}}^{\left|l\right|p}\left(r,z\right)\;\exp\left[i\left(kz+l\phi -k^{\prime }\cdot r\right)\right]{\mathrm{e̅}}_{i}^{\prime }\times \left[e_{k}\alpha_{ik}+A_{ikl}D_{kl}-ik_{l}^{\prime }e_{k}A_{kil}\right]$$

The E1E1–E1E2 interference is therefore

29 |Mfi|αA2=2Ω4n|fLG|l|p(r,z)|2Re[e̅i′ej′ekαik{AjmlD̅ml+ikl′e̅mAmjl}]

Applying the fifth-rank isotropic rotational average^32^ gives

30 ⟨|Mfi|2⟩αA=Ω4n15|fLG|l|p(r,z)|2Re[ϵλπναλσAπνσHA]

where

31 $$H_{A}={\mathrm{D̅}}_{ml}\left[{\mathrm{c̅}}_{m}^{\prime }e_{l}+{\mathrm{c̅}}_{l}^{\prime }e_{m}+{\mathrm{e̅}}_{l}^{\prime }{\left(e\times e^{\prime }\right)}_{m}+{\mathrm{e̅}}_{m}^{\prime }{\left(e\times e^{\prime }\right)}_{l}\right]+i\left(e\cdot k^{\prime }\right)\left(c^{\prime }\cdot \mathrm{e̅}\right)+i\left(e\cdot e^{\prime }\right)\left[\left(k^{\prime }\times {\mathrm{e̅}}^{\prime }\right)\cdot \mathrm{e̅}\right]+i\left({\mathrm{e̅}}^{\prime }\cdot k^{\prime }\right)\left(c\cdot e^{\prime }\right)+i\left({\mathrm{e̅}}^{\prime }\cdot e^{\prime }\right)\left(c\cdot k^{\prime }\right)$$

in which **c** = **e** × **e̅**, **c**′ = **e**′ × **e̅**′, and **c̅**′ = **e̅**′ × **e**′. For a transverse detected plane wave, **e̅**′·**k**′ = 0, so the penultimate term in eq 31 vanishes identically. The use of the full gradient tensor *D*_*ml*_ makes eq 31 valid for the complete T_0_ + L_1_ + T_2_ incident field retained through *O*(ϵ^2^). Its explicit reduction for the detection geometries considered below is given in the Supporting Information. The detected scattered intensity is the energy emitted into the scattered mode per unit time per unit solid angle. Applying Fermi’s golden rule to the second-order Raman amplitude and restoring the final photon density of states gives

32 $$I\left(k^{\prime }\right)\equiv \frac{dP}{d\Omega^{\prime }}=\frac{k^{\prime 3}VN}{4\pi^{2}\hbar }\left\langle {\left|M_{fi}\right|}^{2}\right\rangle$$

where *N* is the number of molecules contributing incoherently to the scattered signal. We define the incident-mode power scale as

33 $$I_{\mathrm{inc}}=\frac{n\hbar c^{2}k}{V{A_{\left|l\right|,p}}^{2}}$$

such that the corresponding leading local energy density and irradiance are *I*_inc_|ψ_*lp*_|^2^/*c* and *I*_inc_|ψ_*lp*_|^2^, respectively.

To expose the cleanest *l*-dependent, σ-independent ROA mechanism, we take linearly polarized input and parallel-analyzed backscattering:

34 $$\begin{array}{l} t=\cos\chi \mathrm{x̂}+\sin\chi ŷ,,u=ẑ\times t,\\ {\mathrm{k̂}}^{\prime }=-ẑ,,{e^{\prime }}_{∥}=t,,{b^{\prime }}_{∥}=-u \end{array}$$

where χ denotes the incident linear-polarization azimuth in the transverse *xy* plane measured from the *x* axis. As established by the order counting above, the conventional T_0_–T_0_ chiral contributions vanish in this linear-polarization channel, so the leading nonzero *l*-odd response occurs at *O*(ϵ^2^). The topological-charge differential is

35 $$\Delta I_{l}^{∥}=I_{∥}\left(+l\right)-I_{∥}\left(-l\right),,l>0$$

For this geometry it is useful to define

36 $$\partial_{t}=t\cdot \nabla_{⊥},,\partial_{u}=u\cdot \nabla_{⊥}$$

and the two beam factors whose *l*-odd parts enter the topological-charge differential,

37 Wlp=−1k2Im[(∂tψlp)(∂uψ̅lp)]Ulp(χ)=1k2Im[(∂t∂uψlp)ψ̅lp]

Strictly, *W*_*lp*_ is odd under *l* → −*l* at arbitrary *z*, whereas *U*_*lp*_^(χ)^ as written is purely *l*-odd only in the beam-waist plane. The explicit parallel-channel results below are therefore evaluated at *z* = 0. Away from the waist, the wavefront-curvature phase generally introduces an *l*-even contribution to *U*_*lp*_^(χ)^, which must be removed by taking its explicit *l*-odd projection when constructing Δ*I*_*l*_^∥^. The first is the L_1_–L_1_ contribution; the second arises from interference of T_0_ with the cross-polarized vectorial part of T_2_. The co-polarized scalar S_2_ part of the T_2_ correction does not enter this parallel chiral signal at *O*(ϵ^2^), although it is retained in the complete field and in the Supporting Information. At the beam waist, writing *f*_*lp*_(*r*) = *f*_LG_^|*l*|*p*^(*r*, 0) and *g*_*lp*_ = *f*_*lp*_^′^/*f*_*lp*_,

38 $$\begin{array}{l} W_{lp}\left(r,0\right)={\left|f_{lp}\left(r\right)\right|}^{2}\frac{l}{k^{2}r}g_{lp}\left(r\right)\\ U_{lp}^{\left(\chi \right)}\left(r,\phi ,0\right)={\left|f_{lp}\left(r\right)\right|}^{2}\frac{l}{k^{2}r}\left(g_{lp}\left(r\right)-\frac{1}{r}\right)\;\cos\left[2\left(\phi -\chi \right)\right] \end{array}$$

Thus, *U*_*lp*_^(χ)^ is local and polarization-aligned, with zero complete azimuthal average.

The full E1M1 contraction is not exhausted by Im­(**E** ·B*), the field pseudoscalar associated with ordinary optical chirality.^38^ Although the T_0_–T_2_ pair cancels from that pseudoscalar, the other isotropic Raman contractions activate a second molecular combination. Electric-quadrupole chiral responses instead contain explicit field-gradient factors,^39,40^ so the same cancellation does not apply to E1E2. We define

39 G1=Im[4αλλG̅μμ−2αλμG̅λμ]GROA=Im[−αλλG̅μμ+3αλμG̅λμ]AROA=Re[ϵλπναλσAπνσ]

For the channel-specific reductions below, the common factor |ψ_*lp*_|^2^ multiplying the factored contractions is absorbed into Φ_*G*,∥_^(*l*‑odd)^ and $H_{A,∥}^{\left(l\mathrm{‐odd}\right)}$ , consistent with the convention used in the Supporting Information. The resulting mode-weighted chiral contractions through *O*(ϵ^2^) are therefore

40 ΦG,∥(l‐odd)=WlpGROA+Ulp(χ)G1Re⁡HA,∥(l‐odd)=kWlp−k+3k′2Ulp(χ)

The full contractions leading to eq 40 are given in the Supporting Information. Combining the E1M1 and E1E2 contributions gives the principal local topological-charge differential:

41 $$\Delta I_{l}^{∥}=\frac{NI_{\mathrm{inc}}k^{\prime 4}}{120\pi^{2}{\varepsilon_{0}}^{2}}\left\{\frac{1}{c}\left[W_{lp}G_{\mathrm{ROA}}+U_{lp}^{\left(\chi \right)}G_{1}\right]+\left[kW_{lp}-\frac{k+3k^{\prime }}{2}U_{lp}^{\left(\chi \right)}\right]A_{\mathrm{ROA}}\right\},,l>0$$

Equation 41 contains all *l*-odd chiral contributions in this parallel-analyzed channel through *O*(ϵ^2^) and is the key result of this work. The two beam factors have distinct physical and spatial origins. The rotationally symmetric factor *W*_*lp*_ is generated from the L_1_–L_1_ contribution in E1M1 and from the L_1_∇_⊥_T_0_ contribution in E1E2. By contrast, *U*_*lp*_^(χ)^ originates from T_0_ and T_2_ and carries the twofold angular dependence cos­[2­(ϕ – χ)]. Complete azimuthal averaging therefore removes *U*_*lp*_^(χ)^, while *W*_*lp*_ remains. An important feature of eq 41 is that *W*_*lp*_ has a direct interpretation in terms of the optical chirality of the incident mode. Up to the conventional overall frequency and field-normalization prefactor, its topological-charge-odd part satisfies

42 [|ψlp|2Im(elp·b̅lp)]l‐odd=−Wlp

Consequently, the rotationally symmetric parts of both chiral interference mechanisms are proportional to the same optical-chirality factor: the E1M1 contribution contains $W_{lp}G_{\mathrm{ROA}}$ , whereas the E1E2 contribution contains ${kW}_{lp}A_{\mathrm{ROA}}$ . This common proportionality provides a direct link between the magnetic-dipole and electric-quadrupole contributions to vortex ROA. Because *U*_*lp*_^(χ)^ has zero complete azimuthal average, this common optical-chirality proportionality becomes exact for the entire surviving azimuthally averaged *l*-odd response in the present parallel channel. The additional *U*_*lp*_^(χ)^ contributions show, however, that optical chirality alone does not exhaust the complete local response with focused vortex beams.

The spatial distributions of the two factors are shown in Figure 3. Reversing *l* reverses the sign of both *W*_*lp*_ and *U*_*lp*_^(χ)^, while their radial and angular structures are controlled by the LG mode indices and the incident linear-polarization direction. The relative contributions of the two factors to the measured ROA signal are determined by the molecule-specific invariants $G_{1}$ , $G_{\mathrm{ROA}}$ , and $A_{\mathrm{ROA}}$ in eq 41. Although *W*_*lp*_ survives complete azimuthal averaging, it also vanishes after uniform radial collection over the entire transverse plane. Complete, uniformly weighted transverse-plane collection therefore gives zero net *l*-odd signal. A nonzero collected response requires incomplete or spatially weighted transverse collection. Finite-aperture, confocal, annular, or spatially resolved Raman detection can additionally select regions in which the local differential signal has a common sign.^41,42^

**Figure 3:**
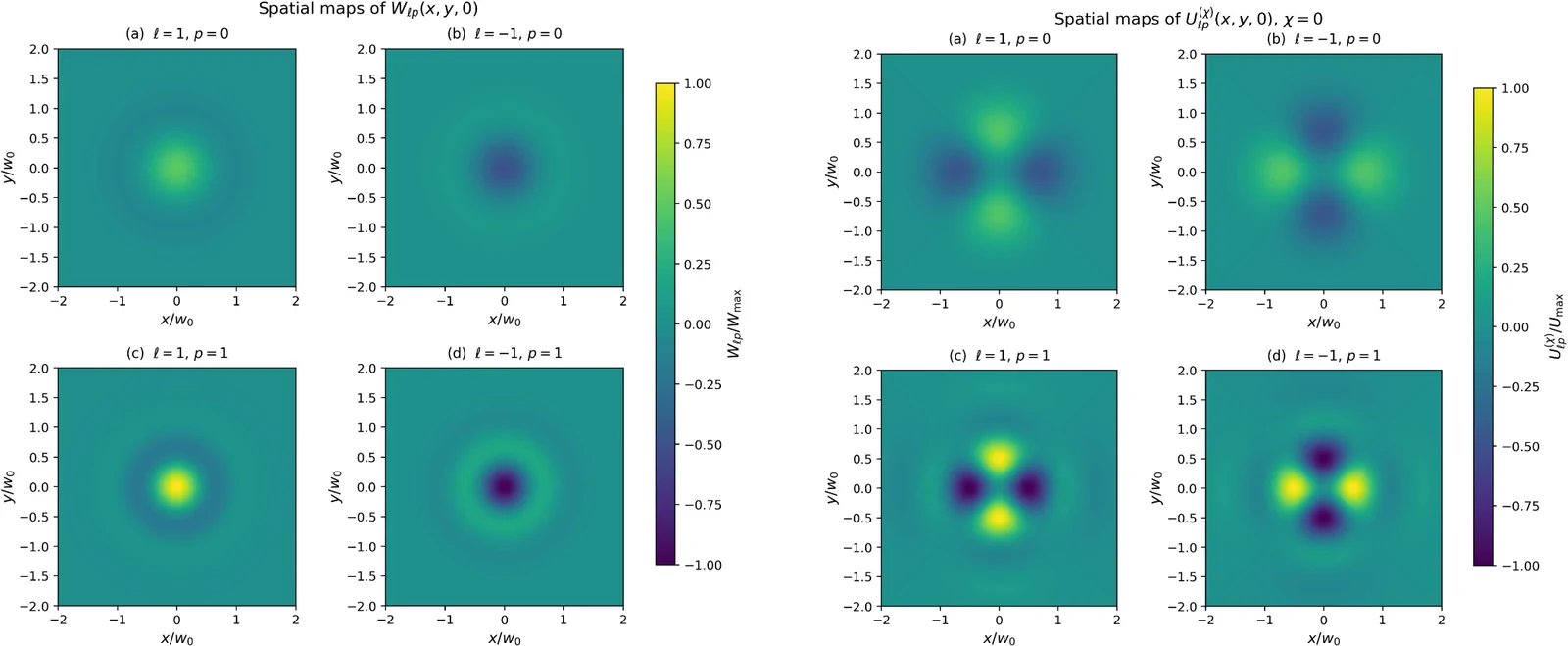
Spatial structure of the two beam-dependent factors controlling the parallel-analyzed vortex ROA signal at the beam waist for *w*_0_ = 3*λ*. Left: the rotationally symmetric longitudinal factor *W*_*lp*_. Right: the second-order transverse factor *U*_*lp*_^(χ)^, shown for χ = 0, whose twofold angular structure follows cos­[2­(ϕ – χ) ]. The panels correspond to (*l*, *p*) = (+1, 0), (−1, 0), (+1, 1), and (−1, 1). Within each set, all panels are normalized to a common maximum absolute value. Reversing the topological charge reverses the sign of both distributions, whereas the complete azimuthal average of *U*_*lp*_^(χ)^ vanishes.

The corresponding radial dependence is shown in Figure 4. For *W*_*lp*_, the radial sign changes follow the amplitude-gradient structure of the LG mode, with increasing *p* introducing additional radial lobes and increasing |*l*| shifting the dominant structure away from the beam axis. The *U*_*lp*_^(χ)^ curves are shown along ϕ = χ, where their angular factor is maximal. Away from this direction the same radial structure is multiplied by cos­[2­(ϕ – χ) ].

**Figure 4:**
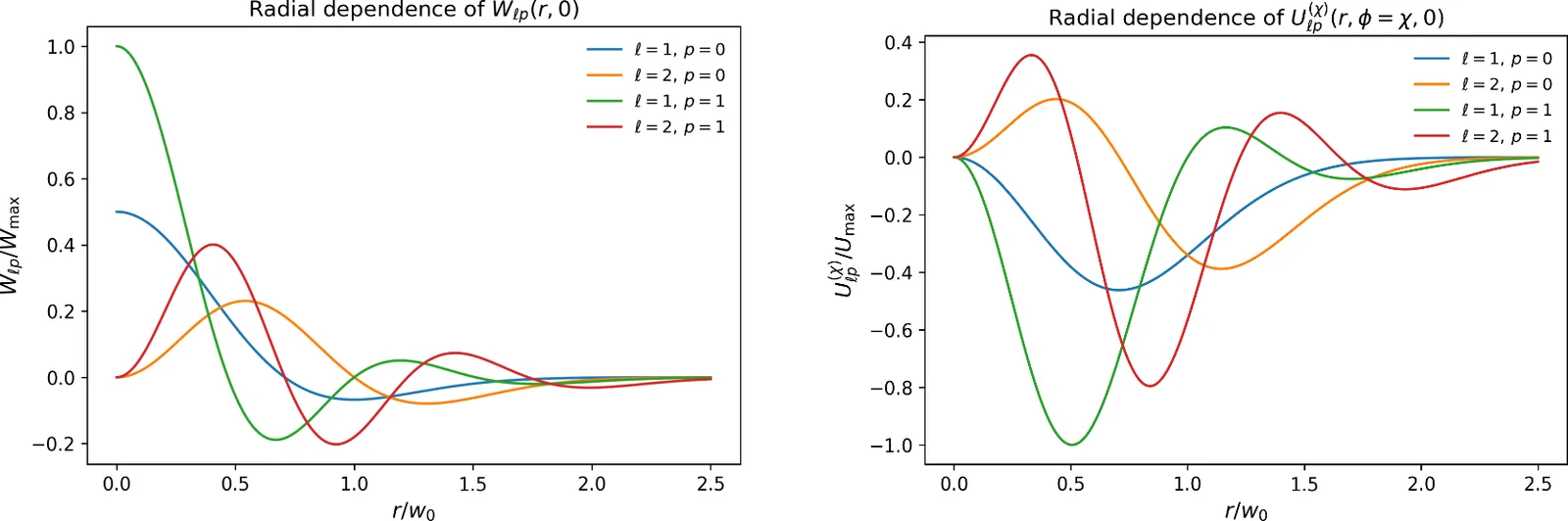
Radial dependence of the beam-dependent factors *W*_*lp*_ (left) and *U*_*lp*_^(χ)^ (right) at the beam waist for *w*_0_ = 3*λ*, *l* = 1, 2, and *p* = 0, 1. The *U*_*lp*_^(χ)^ curves are evaluated along ϕ = χ, for which cos­[2­(ϕ – χ) ] = 1. The radial coordinate is scaled by the beam waist *w*_0_, and each family is normalized to its own common maximum absolute value.

These spatial properties suggest a direct experimental implementation using standard Raman-microscopy components. LG modes with opposite topological charges can be generated at fixed power and linear polarization using a spatial light modulator or vortex phase plates. A chiral solution could then be measured in backscattering with a parallel analyzer and spatially resolved detection selecting regions over which the predicted *l*-odd signal has a common sign. Repeating the measurement for opposite enantiomers would provide an additional control. Raman scattering using vortex beams has already been demonstrated in quartz and chiral liquid crystals, while stimulated Raman scattering has been used to detect optical OAM.^15,43,44^ These experiments establish the practical feasibility of vortex-beam Raman spectroscopy.

If the scattered polarization is not analyzed, the measured signal is *I*_unres_ = *I*_*s*_ + *I*_*p*_, with θ = cos^–1^(**k̂**′·**ẑ**) . The complete local *O*(ϵ^2^) result contains additional *U*_*lp*_^(χ)^- and cos 2ϕ-weighted terms and is given in the Supporting Information. These terms permit a nonzero local E1E2 response in forward and backward scattering but vanish under complete azimuthal averaging. After that average, the E1E2 contribution reduces to

43 $${\overset{®}{\Delta I}}_{l,\alpha A}^{\mathrm{unres},\phi }=\frac{NI_{\mathrm{inc}}k^{\prime 4}}{120\pi^{2}{\varepsilon_{0}}^{2}}W_{lp}kA_{\mathrm{ROA}}\left(\cos^{2}\chi -\sin^{2}\chi \right){\;\sin}^{2}\theta$$

The factor sin^2^ θ shows that this azimuthally averaged, polarization-unresolved *l*-odd E1E2 contribution vanishes in exact forward and backward scattering and is maximal at θ = *π*/2. This angular dependence is characteristic of the vortex-dependent E1E2 channel and is distinct from the more general angular dependence of conventional plane-wave ROA.^25,45^ The complete local response through *O*(ϵ^2^) also contains *U*_*lp*_^(χ)^-weighted terms that need not vanish in the forward or backward directions.

In conclusion, we have shown that Raman optical activity can be controlled by the orbital angular momentum of a focused optical vortex, rather than only by the spin angular momentum of circularly polarized light. The electromagnetic field is retained consistently through T_0_ + L_1_ + T_2_: the longitudinal L_1_ fields generate the rotationally symmetric *W*_*lp*_ contribution, while the *O*(ϵ^2^) transverse completion produces the local *U*_*lp*_^(χ)^ correction. Together with the E1M1 molecular invariants $G_{\mathrm{ROA}}$ and $G_{1}$ and the E1E2 invariant $A_{\mathrm{ROA}}$ , these terms give an *l*-odd Raman response for linearly polarized input from an isotropic ensemble of chiral molecules.

The measured response is selected by polarization analysis, scattering angle, spatially selective detection, and the incident linear polarization. The effect is therefore a polarization-, geometry-, and spatially resolved multipolar Raman interference signal, establishing optical topological charge as a control parameter for ROA beyond the conventional circular-polarization paradigm.

## Supplementary Material



## Notes

Additional derivations of the complete T_0_ + L_1_ + T_2_ electromagnetic field, order counting in the paraxiality parameter, LG field gradients, rotational averages, scattering contractions, rate prefactors, and detection-channel reductions (PDF)
