# Supplementary material for: Twisted Raman: Orbital Angular Momentum Control of Raman Optical Activity
Source: J Phys Chem Lett. 2026 Sep 6;17(37):10682–90. doi: 10.1021/acs.jpclett.6c02215 (PMC13596026; doi:10.1021/acs.jpclett.6c02215)
Supplement: Supplementary file 1 [file jz6c02215_si_001.pdf]

Supporting Information: Twisted Raman: Orbital Angular Momentum Control of Raman Optical Activity

Kayn A. Forbes*

School of Chemistry, University of East Anglia, Norwich Research Park, Norwich NR4 7TJ, United Kingdom

Notation, Laguerre–Gaussian fields, and paraxial-order bookkeeping

This Supporting Information gives the algebra leading from the focused Laguerre–Gaussian (LG) fields to the polarisation-resolved Raman and Raman optical activity (ROA) scattering rates. Repeated Cartesian indices are summed and an overbar denotes complex conjugation. The incident field is retained consistently through the zeroth-order transverse field T_0, first-order longitudinal field L_1, and second-order transverse field T_2.

The scalar LG mode is written

$$\psi_{\ell p}(\mathbf{r}) = f_{\mathrm{LG}}^{|\ell|p}(r,z)\exp[i(kz+\ell\phi)], \tag{1}$$

with

$$\begin{aligned} f_{\mathrm{LG}}^{|\ell|p}(r,z) = \mathcal{N}_{\ell p}\frac{w_0}{w(z)}\left(\frac{\sqrt{2}r}{w(z)}\right)^{|\ell|} L_p^{|\ell|}\left(\frac{2r^2}{w^2(z)}\right)\exp\left[-\frac{r^2}{w^2(z)}\right] \\ \times \exp i\left[\frac{kr^2}{2R(z)} - (2p+|\ell|+1)\zeta(z)\right], \end{aligned} \tag{2}$$

$$\mathcal{N}_{\ell p} = \left[\frac{2p!}{\pi w_0^2(p+|\ell|)!}\right]^{1/2}, \tag{3}$$

where

$$w(z) = w_0\left(1+\frac{z^2}{z_R^2}\right)^{1/2}, \qquad R(z) = z\left(1+\frac{z_R^2}{z^2}\right), \qquad \zeta(z) = \tan^{-1}\left(\frac{z}{z_R}\right). \tag{4}$$

The logarithmic radial derivative is

$$\begin{aligned} \gamma_{|\ell|p} &= \frac{1}{f_{\mathrm{LG}}^{|\ell|p}}\frac{\partial f_{\mathrm{LG}}^{|\ell|p}}{\partial r} \\ &= \frac{|\ell|}{r} - \frac{2r}{w^2} + \frac{ikr}{R} - \frac{4r}{w^2}\frac{L_{p-1}^{|\ell|+1}(2r^2/w^2)}{L_p^{|\ell|}(2r^2/w^2)}, \end{aligned} \tag{5}$$

where the final term is absent for $p = 0$. The transverse derivatives are

$$Q_{\ell p} = \gamma_{|\ell|p} \cos\phi - \frac{i\ell}{r} \sin\phi, \qquad P_{\ell p} = \gamma_{|\ell|p} \sin\phi + \frac{i\ell}{r} \cos\phi, \tag{6}$$

so that

$$\partial_x \psi_{\ell p} = Q_{\ell p} \psi_{\ell p}, \qquad \partial_y \psi_{\ell p} = P_{\ell p} \psi_{\ell p}. \tag{7}$$

The exact longitudinal logarithmic derivative is

$$\mathcal{Z}_{|\ell|p} = \frac{1}{\psi_{\ell p}} \frac{\partial \psi_{\ell p}}{\partial z} = ik + \delta Z_{\ell p}, \tag{8}$$

where

$$\delta Z_{\ell p} \equiv \frac{1}{f_{\mathrm{LG}}^{|\ell|p}} \frac{\partial f_{\mathrm{LG}}^{|\ell|p}}{\partial z} = \frac{\partial}{\partial z} \ln f_{\mathrm{LG}}^{|\ell|p} = O(k\epsilon^2), \qquad \epsilon \equiv (kw_0)^{-1}. \tag{9}$$

Accordingly,

$$\begin{aligned} \mathbf{T}^{\mathrm{full}} &= Q_{\ell p} \hat{\mathbf{x}} + P_{\ell p} \hat{\mathbf{y}} + (ik + \delta Z_{\ell p})\, \hat{\mathbf{z}} \\ &= \mathbf{T} + \delta Z_{\ell p} \hat{\mathbf{z}}, \\ \mathbf{T} &= Q_{\ell p} \hat{\mathbf{x}} + P_{\ell p} \hat{\mathbf{y}} + ik\hat{\mathbf{z}}. \end{aligned} \tag{10}$$

Here $\mathbf{T}^{\mathrm{full}}$ is the exact scalar-mode gradient factor, whereas $\mathbf{T}$ retains only the leading carrier contribution to its longitudinal component. The incident transverse Jones state and its 90° rotation are

$$\mathbf{t} = \alpha\hat{\mathbf{x}} + \beta\hat{\mathbf{y}}, \qquad \mathbf{u} = \hat{\mathbf{z}} \times \mathbf{t} = -\beta\hat{\mathbf{x}} + \alpha\hat{\mathbf{y}}, \qquad |\alpha|^2 + |\beta|^2 = 1. \tag{11}$$

Directional derivatives are

$$\partial_t = \mathbf{t} \cdot \nabla_\perp, \qquad \partial_u = \mathbf{u} \cdot \nabla_\perp. \tag{12}$$

Dual-symmetrized $\mathrm{T}_0 + \mathrm{L}_1 + \mathrm{T}_2$ field

We use the same factored incident-field notation as in the Letter,

$$\mathbf{E}^{(+)}_{\mathrm{LG}} = \Omega\, \mathbf{e}_{\ell p} \psi_{\ell p}\, a_{\ell p}, \qquad \mathbf{B}^{(+)}_{\mathrm{LG}} = \frac{\Omega}{c}\, \mathbf{b}_{\ell p} \psi_{\ell p}\, a_{\ell p}. \tag{13}$$

Through $O(\epsilon^2)$

$$\mathbf{e}_{\ell p}\psi_{\ell p} = \underbrace{\mathbf{t}\psi_{\ell p}}_{\mathrm{T}_0} + \underbrace{\hat{\mathbf{z}}\frac{i}{k}\partial_t \psi_{\ell p}}_{\mathrm{L}_1} + \underbrace{\mathcal{T}_2[\mathbf{t}]\psi_{\ell p}}_{\mathrm{T}_2}, \tag{14}$$

$$\mathbf{b}_{\ell p}\psi_{\ell p} = \underbrace{\mathbf{u}\psi_{\ell p}}_{\mathrm{T}_0} + \underbrace{\hat{\mathbf{z}}\frac{i}{k}\partial_u \psi_{\ell p}}_{\mathrm{L}_1} + \underbrace{\mathcal{T}_2[\mathbf{u}]\psi_{\ell p}}_{\mathrm{T}_2}. \tag{15}$$

In the dual-symmetrized reference-plane convention used for the explicit second-order results,

$$\boxed{\mathcal{T}_2[\mathbf{v}]\psi = \frac{1}{4k^2}\left[\mathbf{v}\, \nabla_\perp^2 \psi + 2\nabla_\perp \left(\mathbf{v} \cdot \nabla_\perp \psi\right)\right].} \tag{16}$$

This may be separated into scalar-envelope and vectorial pieces,

$$\mathcal{T}_2[\mathbf{v}]\psi = \underbrace{\mathbf{v}\frac{\nabla_\perp^2 \psi}{2k^2}}_{\mathrm{S}_2} + \underbrace{\frac{2\nabla_\perp(\mathbf{v} \cdot \nabla_\perp \psi) - \mathbf{v}\nabla_\perp^2 \psi}{4k^2}}_{\mathrm{V}_2}. \tag{17}$$

Thus T_2 is not an independent mode: it is the transverse second-order completion fixed by the same source-free Maxwell field.

Below, the ℓp labels on $\psi_{\ell p}$, $\mathbf{e}_{\ell p}$, and $\mathbf{b}_{\ell p}$ are suppressed where no ambiguity can arise.

For real linear input it is useful to use the orthonormal basis $(\mathbf{t}, \mathbf{u}, \hat{\mathbf{z}})$. Then

$$\mathbf{e}^{\mathrm{T}_0}\psi = \mathbf{t}\psi, \qquad \mathbf{e}^{\mathrm{L}_1}\psi = \hat{\mathbf{z}}\frac{i}{k}\psi_t,$$
$$\mathbf{e}^{\mathrm{T}_2}\psi = \mathbf{t}\left[\psi^{(\mathrm{S}_2)} + \frac{\psi_{tt} - \psi_{uu}}{4k^2}\right] + \mathbf{u}\frac{\psi_{tu}}{2k^2}, \tag{18}$$
$$\mathbf{b}^{\mathrm{T}_0}\psi = \mathbf{u}\psi, \qquad \mathbf{b}^{\mathrm{L}_1}\psi = \hat{\mathbf{z}}\frac{i}{k}\psi_u,$$
$$\mathbf{b}^{\mathrm{T}_2}\psi = \mathbf{u}\left[\psi^{(\mathrm{S}_2)} - \frac{\psi_{tt} - \psi_{uu}}{4k^2}\right] + \mathbf{t}\frac{\psi_{tu}}{2k^2}, \tag{19}$$

where

$$\psi_t = \partial_t\psi, \qquad \psi_u = \partial_u\psi, \qquad \psi_{tu} = \partial_t\partial_u\psi, \qquad \psi^{(\mathrm{S}_2)} = \frac{\psi_{tt} + \psi_{uu}}{2k^2}. \tag{20}$$

The cross-polarised V_2 projection relevant to the Letter is therefore

$$\mathbf{u}\cdot\mathbf{e}^{\mathrm{T}_2}\psi = \mathbf{t}\cdot\mathbf{b}^{\mathrm{T}_2}\psi = \frac{\psi_{tu}}{2k^2}. \tag{21}$$

Dividing Eqs. (14) and (15) by $\psi_{\ell p}$ gives the dimensionless local factors used in the Letter,

$$\mathbf{e}_{\ell p} = \mathbf{t} + \frac{i}{k}S_{\ell p}\hat{\mathbf{z}} + \mathbf{e}^{\mathrm{T}_2}_{\ell p},$$
$$\mathbf{b}_{\ell p} = \mathbf{u} + \frac{i}{k}R_{\ell p}\hat{\mathbf{z}} + \mathbf{b}^{\mathrm{T}_2}_{\ell p}, \tag{22}$$

where

$$S_{\ell p} = \alpha Q_{\ell p} + \beta P_{\ell p}, \qquad R_{\ell p} = \alpha P_{\ell p} - \beta Q_{\ell p}. \tag{23}$$

Equations (14)–(15) are preferable at radial nodes because they do not divide by $\psi_{\ell p}$.

Complete electric-field gradient

The E1E2 interaction requires the gradient of the *complete* incident electric field. In the same factored notation as the Letter, define

$$\begin{aligned} D_{ml}\psi_{\ell p} &= \nabla_l \left(e_{\ell p,m}\psi_{\ell p} \right) \\ &= \left[t_m T_l^{\text{full}} + \frac{i}{k}\hat{z}_m L_l + D_{ml}^{\mathrm{T}_2} \right] \psi_{\ell p} \\ &= \left[t_m T_l + t_m \hat{z}_l \, \delta Z_{\ell p} + \frac{i}{k}\hat{z}_m L_l + D_{ml}^{\mathrm{T}_2} \right] \psi_{\ell p}. \end{aligned} \tag{24}$$

where

$$\nabla_l (S_{\ell p}\psi_{\ell p}) = L_l \psi_{\ell p}, \qquad D_{ml}^{\mathrm{T}_2}\psi_{\ell p} = \nabla_l \left(e_{\ell p,m}^{\mathrm{T}_2}\psi_{\ell p} \right). \tag{25}$$

The term $t_m \hat{z}_l \, \delta Z_{\ell p}$ is the second-order longitudinal-envelope derivative of the T_0 field. It is even under $\ell \to -\ell$ and therefore does not contribute to the topological-charge differential, but it is retained here so that D_{ml} represents the complete field gradient through $O(\epsilon^2)$.

For completeness,

$$L_l = \nabla_l S_{\ell p} + S_{\ell p} \nabla_l \ln \psi_{\ell p}. \tag{26}$$

The leading carrier reduction is

$$\mathbf{L} \to ik S_{\ell p} \hat{\mathbf{z}}. \tag{27}$$

The full D_{ml} representation will be used below so that the T_2 gradient is never omitted by construction.

Smallness-parameter bookkeeping

With

$$\epsilon = (kw_0)^{-1}, \tag{28}$$

the field hierarchy is

$$\mathrm{T}_0 = O(1), \qquad \mathrm{L}_1 = O(\epsilon), \qquad \mathrm{T}_2 = O(\epsilon^2). \tag{29}$$

For arbitrary elliptical or circular polarisation, the conventional leading ROA response is the T_0–T_0 chiral interference contribution, which is of zeroth order in ϵ. In the E1E2 channel, the notation T_0–T_0 includes the carrier-gradient contribution $\partial_z \mathrm{T}_0 \simeq ik\mathrm{T}_0$, which does not introduce an additional power of ϵ. This leading contribution is therefore generally present and is not being neglected here.

For the real linearly polarised incident field and linear analysed output used for the principal channel in the Letter, however, the zeroth-order transverse polarisation factors do not generate a chiral E1M1 or E1E2 contraction. Consequently, the T_0–T_0 chiral contributions vanish in that channel. In addition, the zeroth-order transverse field is independent of the sign of ℓ, so any T_0–T_0 contribution would cancel from the fixed-polarisation topological-charge differential $I(+\ell) - I(-\ell)$. The leading nonzero ℓ-odd response therefore occurs at total order $O(\epsilon^2)$, requiring all contributions involving L_1 and T_2 at that order to be retained consistently.

The gradient hierarchy is

$$\begin{aligned} \partial_z \mathrm{T}_0 &= O(k), & \nabla_\perp \mathrm{T}_0,\ \partial_z \mathrm{L}_1 &= O(k\epsilon), \\ \nabla_\perp \mathrm{L}_1,\ \partial_z \mathrm{T}_2,\ \partial_z^{\mathrm{env}} \mathrm{T}_0 &= O(k\epsilon^2), & \nabla_\perp \mathrm{T}_2 &= O(k\epsilon^3). \end{aligned} \tag{30}$$

The carrier derivative $\partial_z\psi \simeq ik\psi$ therefore does not add a power of ϵ. In E1M1,

$$\mathrm{L}_1\,\mathrm{L}_1 \sim \mathrm{T}_0\,\mathrm{T}_2 \sim O(\epsilon^2), \tag{31}$$

whereas in E1E2,

$$\mathrm{L}_1\,\nabla_\perp\mathrm{T}_0 \sim \mathrm{T}_2\,\partial_z\mathrm{T}_0 \sim \mathrm{T}_0\,\partial_z\mathrm{T}_2 \sim O(k\epsilon^2). \tag{32}$$

At amplitude level this is the statement

$$M_\alpha^* M_X\big|_{O(\epsilon^2)} = M_\alpha^{(0)*}M_X^{(2)} + M_\alpha^{(1)*}M_X^{(1)} + M_\alpha^{(2)*}M_X^{(0)}, \tag{33}$$

with $X = G$ or A. Terms must therefore be assembled at a fixed ϵ order before cancellations associated with a particular polarisation or molecular contraction are imposed.

Electric-dipole–electric-dipole Raman scattering

Let Ω^2 denote the product of the incident and scattered single-photon electric-field amplitudes. Suppressing the ℓp labels on $\mathbf{e}_{\ell p}$ and $\psi_{\ell p}$, as in the Letter,

$$M_{fi}^{(\alpha)} = -\Omega^2\sqrt{n}\,\psi_{\ell p}\,\bar{e}_i'e_k\alpha_{ik}e^{-i\mathbf{k}'\cdot\mathbf{r}}. \tag{34}$$

The squared amplitude is

$$|M_{fi}^{(\alpha)}|^2 = n\Omega^4|\psi_{\ell p}|^2\bar{e}_i'e_j'e_k\bar{e}_l\alpha_{ik}\bar{\alpha}_{jl}. \tag{35}$$

For two second-rank tensors,

$$\langle A_{ij}B_{kl}\rangle = \frac{1}{30}\Big[\delta_{ij}\delta_{kl}(4I_1 - I_2 - I_3) + \delta_{ik}\delta_{jl}(-I_1 + 4I_2 - I_3)$$

$$+ \delta_{il}\delta_{jk}(-I_1 - I_2 + 4I_3)\Big], \tag{36}$$

where

$$I_1 = A_{\lambda\lambda}B_{\mu\mu}, \quad I_2 = A_{\lambda\mu}B_{\lambda\mu}, \quad I_3 = A_{\lambda\mu}B_{\mu\lambda}. \tag{37}$$

Defining

$$A_1 = \alpha_{\lambda\lambda}\bar{\alpha}_{\mu\mu}, \qquad A_2 = \alpha_{\lambda\mu}\bar{\alpha}_{\lambda\mu}, \qquad A_3 = \alpha_{\lambda\mu}\bar{\alpha}_{\mu\lambda}, \tag{38}$$

the isotropic E1E1 contribution is

$$\begin{aligned}\left\langle |M_{fi}^{(\alpha)}|^2 \right\rangle = \frac{n\Omega^4}{30}|\psi_{\ell p}|^2 \Big[&(\bar{\mathbf{e}}' \cdot \mathbf{e})(\mathbf{e}' \cdot \bar{\mathbf{e}})(4A_1 - A_2 - A_3) \\ &+ (\bar{\mathbf{e}}' \cdot \mathbf{e}')(\mathbf{e} \cdot \bar{\mathbf{e}})(-A_1 + 4A_2 - A_3) \\ &+ (\bar{\mathbf{e}}' \cdot \bar{\mathbf{e}})(\mathbf{e} \cdot \mathbf{e}')(-A_1 - A_2 + 4A_3)\Big].\end{aligned} \tag{39}$$

Because $\mathbf{e}_{\ell p}\psi_{\ell p}$ in Eq. (14) contains $\mathrm{T}_0 + \mathrm{L}_1 + \mathrm{T}_2$, Eq. (39) includes both $\mathrm{L}_1\,\mathrm{L}_1$ and $\mathrm{T}_0\,\mathrm{T}_2$ corrections to the achiral Raman baseline through $O(\epsilon^2)$.

Electric-dipole–magnetic-dipole ROA contribution

Using the same local incident factors $\mathbf{e}_{\ell p}$ and $\mathbf{b}_{\ell p}$ as the Letter, the E1E1+E1M1 amplitude is

$$M_{fi}^{(\alpha+G)} = -\Omega^2\sqrt{n}\,\psi_{\ell p}e^{-i\mathbf{k}'\cdot\mathbf{r}}\left[\bar{e}_i'e_j\alpha_{ij} + \frac{1}{c}\bar{e}_i'b_jG_{ij} + \frac{1}{c}\bar{b}_i'e_j\bar{G}_{ji}\right]. \tag{40}$$

The E1E1–E1M1 interference is

$$|M_{fi}|^2_{\alpha G} = \frac{2n\Omega^4}{c}|\psi_{\ell p}|^2\,\mathrm{Re}\left[\bar{e}_i'e_je_k'\bar{b}_l\alpha_{ij}\bar{G}_{kl} + \bar{e}_i'e_jb_k'\bar{e}_l\alpha_{ij}G_{lk}\right]. \tag{41}$$

In the non-resonant Raman limit $\alpha_{ij} = \alpha_{ji}$, define

$$\mathcal{G}_1^- = 4\alpha_{\lambda\lambda}\bar{G}_{\mu\mu} - 2\alpha_{\lambda\mu}\bar{G}_{\lambda\mu}, \qquad \mathcal{G}_2^- = -\alpha_{\lambda\lambda}\bar{G}_{\mu\mu} + 3\alpha_{\lambda\mu}\bar{G}_{\lambda\mu},$$
$$\mathcal{G}_1^+ = 4\alpha_{\lambda\lambda}G_{\mu\mu} - 2\alpha_{\lambda\mu}G_{\lambda\mu}, \qquad \mathcal{G}_2^+ = -\alpha_{\lambda\lambda}G_{\mu\mu} + 3\alpha_{\lambda\mu}G_{\lambda\mu}. \tag{42}$$

The fourth-rank isotropic average gives

$$\left\langle |M_{fi}|^2 \right\rangle_{\alpha G} = \frac{2n\Omega^4}{30c}|\psi_{\ell p}|^2 \,\mathrm{Re}\left[F_1\mathcal{G}_1^- + F_2\mathcal{G}_2^- + F_3\mathcal{G}_2^- + F_4\mathcal{G}_1^+ + F_5\mathcal{G}_2^+ + F_6\mathcal{G}_2^+\right], \tag{43}$$

where

$$F_1 = (\bar{\mathbf{e}}' \cdot \mathbf{e})(\mathbf{e}' \cdot \bar{\mathbf{b}}), \qquad F_2 = (\bar{\mathbf{e}}' \cdot \mathbf{e}')(\mathbf{e} \cdot \bar{\mathbf{b}}),$$
$$F_3 = (\bar{\mathbf{e}}' \cdot \bar{\mathbf{b}})(\mathbf{e} \cdot \mathbf{e}'), \qquad F_4 = (\bar{\mathbf{e}}' \cdot \mathbf{e})(\mathbf{b}' \cdot \bar{\mathbf{e}}),$$
$$F_5 = (\bar{\mathbf{e}}' \cdot \mathbf{b}')(\mathbf{e} \cdot \bar{\mathbf{e}}), \qquad F_6 = (\bar{\mathbf{e}}' \cdot \bar{\mathbf{e}})(\mathbf{e} \cdot \mathbf{b}'). \tag{44}$$

These are the same six isotropic contractions as in the Letter, but the incident fields are now explicitly the complete $\mathrm{T}_0 + \mathrm{L}_1 + \mathrm{T}_2$ fields.

For a non-resonant response $\mathcal{G}_a^+ = (\mathcal{G}_a^-)^*$. The real molecular invariants used in the final linearly polarised result are

$$\mathcal{G}_1 = \mathrm{Im}\,\mathcal{G}_1^- = \mathrm{Im}\left[4\alpha_{\lambda\lambda}\bar{G}_{\mu\mu} - 2\alpha_{\lambda\mu}\bar{G}_{\lambda\mu}\right],$$
$$\mathcal{G}_{\mathrm{ROA}} = \mathrm{Im}\,\mathcal{G}_2^- = \mathrm{Im}\left[-\alpha_{\lambda\lambda}\bar{G}_{\mu\mu} + 3\alpha_{\lambda\mu}\bar{G}_{\lambda\mu}\right]. \tag{45}$$

Electric-dipole–electric-quadrupole ROA contribution

The electric-quadrupole operator convention is the one used in the Letter,

$$H_{E2} = -Q_{ij}\nabla_j E_i, \tag{46}$$

so no additional numerical factor is hidden when E1M1 and E1E2 contributions are combined.

Using the full factored gradient D_{ml} defined in Eq. (24), the E1E1+E1E2 amplitude is

$$M_{fi}^{(\alpha+A)} = -\Omega^2\sqrt{n}\,\psi_{\ell p}\bar{e}_i'\left[e_k\alpha_{ik} + A_{ikl}D_{kl} - ik_l'e_kA_{kil}\right]e^{-i\mathbf{k}'\cdot\mathbf{r}}. \tag{47}$$

The interference is

$$|M_{fi}|^2_{\alpha A} = 2n\Omega^4|\psi_{\ell p}|^2\,\mathrm{Re}\left[\bar{e}_i'e_j'e_k\alpha_{ik}\left\{A_{jml}\bar{D}_{ml} + ik_l'\bar{e}_mA_{mjl}\right\}\right]. \tag{48}$$

Define

$$\mathcal{A}_{\alpha A} = \epsilon_{\lambda\pi\nu}\alpha_{\lambda\sigma}A_{\pi\nu\sigma}. \tag{49}$$

For $\alpha_{ij} = \alpha_{ji}$ and $A_{i,jk} = A_{i,kj}$, the two required fifth-rank averages are

$$\langle\alpha_{ik}A_{jml}\rangle = \frac{\mathcal{A}_{\alpha A}}{30}\left(\epsilon_{ijm}\delta_{kl} + \epsilon_{ijl}\delta_{km} + \epsilon_{kjm}\delta_{il} + \epsilon_{kjl}\delta_{im}\right), \tag{50}$$

$$\langle\alpha_{ik}A_{mjl}\rangle = \frac{\mathcal{A}_{\alpha A}}{30}\left(\epsilon_{imj}\delta_{kl} + \epsilon_{iml}\delta_{kj} + \epsilon_{kmj}\delta_{il} + \epsilon_{kml}\delta_{ij}\right). \tag{51}$$

Thus

$$\left\langle|M_{fi}|^2\right\rangle_{\alpha A} = \frac{2n\Omega^4}{30}|\psi_{\ell p}|^2\,\mathrm{Re}\left[\mathcal{A}_{\alpha A}\mathcal{H}_A\right], \tag{52}$$

where, for an arbitrary normalized transverse analysed state $\mathbf{e}'$,

$$\begin{aligned}\mathcal{H}_A = \bar{D}_{ml}&\Big[\bar{c}_m'e_l + \bar{c}_l'e_m + \bar{e}_l'(\mathbf{e}\times\mathbf{e}')_m + \bar{e}_m'(\mathbf{e}\times\mathbf{e}')_l\Big]\\ &+ i(\mathbf{e}\cdot\mathbf{k}')(\mathbf{c}'\cdot\bar{\mathbf{e}}) + i(\mathbf{e}\cdot\mathbf{e}')\left[(\mathbf{k}'\times\bar{\mathbf{e}}')\cdot\bar{\mathbf{e}}\right]\\ &+ i(\bar{\mathbf{e}}'\cdot\mathbf{e}')(\mathbf{c}\cdot\mathbf{k}'),\end{aligned} \tag{53}$$

with

$$\bar{\mathbf{c}}' = \bar{\mathbf{e}}' \times \mathbf{e}', \qquad \mathbf{c}' = \mathbf{e}' \times \bar{\mathbf{e}}', \qquad \mathbf{c} = \mathbf{e} \times \bar{\mathbf{e}}. \tag{54}$$

The term proportional to $(\bar{\mathbf{e}}' \cdot \mathbf{k}')$ produced by the second fifth-rank average is zero for a physical transverse output state. Equation (53) is algebraically equivalent to the longer T/L-split expression used previously, but it remains valid when T_2 is included and is therefore used throughout this revision.

The molecular E1E2 invariant entering the non-resonant rate is

$$\mathcal{A}_{\mathrm{ROA}} = \mathrm{Re}\left[\epsilon_{\lambda\pi\nu}\alpha_{\lambda\sigma}A_{\pi\nu\sigma}\right]. \tag{55}$$

Molecular response tensors and complete chiral rate

For a Raman transition $|E_i\rangle \to |E_f\rangle$, the transition tensors are

$$\alpha_{\lambda\mu} = \sum_r \left[\frac{\mu_\lambda^{fr}\mu_\mu^{ri}}{E_{ri} - \hbar\omega} + \frac{\mu_\mu^{fr}\mu_\lambda^{ri}}{E_{ri} + \hbar\omega'}\right], \tag{56}$$

$$G_{\lambda\mu} = \sum_r \left[\frac{\mu_\lambda^{fr}m_\mu^{ri}}{E_{ri} - \hbar\omega} + \frac{m_\mu^{fr}\mu_\lambda^{ri}}{E_{ri} + \hbar\omega'}\right], \tag{57}$$

$$A_{\lambda\mu\nu} = \sum_r \left[\frac{\mu_\lambda^{fr}Q_{\mu\nu}^{ri}}{E_{ri} - \hbar\omega} + \frac{Q_{\mu\nu}^{fr}\mu_\lambda^{ri}}{E_{ri} + \hbar\omega'}\right]. \tag{58}$$

The $Q_{\mu\nu}$ in the final line is exactly the quadrupole operator in Eq. (46). The chiral part of the isotropic Raman rate is

$$\Gamma_\chi = \Gamma_{\alpha G} + \Gamma_{\alpha A}, \tag{59}$$

and changes sign on enantiomer reversal. For handedness $h = \pm 1$,

$$\Gamma_h = \Gamma_{\alpha\alpha} + h\Gamma_\chi, \qquad \Gamma_+ - \Gamma_- = 2\Gamma_\chi. \tag{60}$$

From squared matrix elements to physical intensity

The density of final photon states per unit energy and solid angle is

$$\rho_f = \frac{Vk'^2}{(2\pi)^3 \hbar c}. \tag{61}$$

Fermi's golden rule and multiplication by $\hbar c k'$ give

$$I(\mathbf{k}') \equiv \frac{dP}{d\Omega'} = \frac{k'^3 VN}{4\pi^2\hbar} \left\langle |M_{fi}|^2 \right\rangle. \tag{62}$$

For the product of incident LG and scattered plane-wave electric-field normalizations,

$$\Omega^4 = \frac{\hbar^2 c^2 k k'}{4\varepsilon_0^2 V^2 A_{|\ell|,p}^2}. \tag{63}$$

We define the incident mode-power scale $I_{\mathrm{inc}} = n\hbar c^2 k/(VA_{\ell,p}^2)$, such that the corresponding leading local energy density and irradiance are $I_{\mathrm{inc}}|\psi_{\ell p}|^2/c$ and $I_{\mathrm{inc}}|\psi_{\ell p}|^2$, respectively. Hence

$$\frac{k'^3 VN}{4\pi^2\hbar} \frac{2n\Omega^4}{15} = \frac{N I_{\mathrm{inc}} k'^4}{120\pi^2\varepsilon_0^2}. \tag{64}$$

This is the common prefactor used in the revised Letter. The E1M1 rate contains the additional explicit $1/c$ from the magnetic field, whereas the E1E2 rate contains a field gradient and therefore an optical factor of order k.

General scattering geometry

The incident beam propagates along $+\hat{\mathbf{z}}$, and the scattering plane is the yz plane:

$$\hat{\mathbf{k}}' = \sin\theta\,\hat{\mathbf{y}} + \cos\theta\,\hat{\mathbf{z}}, \qquad \mathbf{k}' = k'\hat{\mathbf{k}}'. \tag{65}$$

The linear output basis is

$$\hat{\mathbf{s}} = \hat{\mathbf{x}}, \qquad \hat{\mathbf{p}} = \cos\theta\,\hat{\mathbf{y}} - \sin\theta\,\hat{\mathbf{z}}. \tag{66}$$

A general incident polarisation is parameterised by

$$\alpha = \cos\eta\cos\chi - i\sin\eta\sin\chi, \qquad \beta = \cos\eta\sin\chi + i\sin\eta\cos\chi, \tag{67}$$

Here χ is the azimuth of the incident polarisation ellipse and η is its ellipticity angle. The primed quantities χ' and η' denote the corresponding azimuth and ellipticity angle of the analysed output state. A general analysed output is parameterised by

$$c_s = \cos\eta'\cos\chi' - i\sin\eta'\sin\chi', \qquad c_p = \cos\eta'\sin\chi' + i\sin\eta'\cos\chi', \tag{68}$$

so that

$$\mathbf{e}' = c_s\hat{\mathbf{s}} + c_p\hat{\mathbf{p}}, \qquad \mathbf{b}' = \hat{\mathbf{k}}' \times \mathbf{e}' = -c_p\hat{\mathbf{s}} + c_s\hat{\mathbf{p}}. \tag{69}$$

For arbitrary $(\chi, \eta, \chi', \eta', \theta)$, the polarisation-resolved rates through $O(\epsilon^2)$ are obtained by inserting Eqs. (14), (15), and (24) directly into Eqs. (39), (43), and (53). The resulting products are expanded and truncated consistently at total Lax order $O(\epsilon^2)$; terms of order $O(\epsilon^3)$ and higher generated by multiplying the truncated fields are discarded.

For later use, define the projections

$$e_s = \hat{\mathbf{s}} \cdot \mathbf{e}, \qquad e_p = \hat{\mathbf{p}} \cdot \mathbf{e}, \qquad b_s = \hat{\mathbf{s}} \cdot \mathbf{b}, \qquad b_p = \hat{\mathbf{p}} \cdot \mathbf{b}. \tag{70}$$

Then, for example,

$$\begin{aligned} \bar{\mathbf{e}}' \cdot \mathbf{e} &= \bar{c}_s e_s + \bar{c}_p e_p, & \mathbf{e}' \cdot \bar{\mathbf{b}} &= c_s \bar{b}_s + c_p \bar{b}_p, \\ \mathbf{b}' \cdot \bar{\mathbf{e}} &= -c_p \bar{e}_s + c_s \bar{e}_p, & \bar{\mathbf{e}}' \cdot \mathbf{b}' &= -\bar{c}_s c_p + \bar{c}_p c_s. \end{aligned} \tag{71}$$

Together with the corresponding conjugates, these relations give the six F_a without expanding the T_2 operator into lengthy Cartesian expressions.

Linearly polarised input: parallel analysed backscattering

For the primary Letter observable,

$$\mathbf{t} = \cos\chi\,\hat{\mathbf{x}} + \sin\chi\,\hat{\mathbf{y}}, \qquad \mathbf{u} = -\sin\chi\,\hat{\mathbf{x}} + \cos\chi\,\hat{\mathbf{y}}, \tag{72}$$

and

$$\hat{\mathbf{k}}' = -\hat{\mathbf{z}}, \qquad \mathbf{e}'_{\parallel} = \mathbf{t}, \qquad \mathbf{b}'_{\parallel} = -\mathbf{u}. \tag{73}$$

The topological-charge differential is

$$\Delta I_\ell^{\parallel} = I_{\parallel}(+\ell) - I_{\parallel}(-\ell), \qquad \ell > 0. \tag{74}$$

In the channel-specific reductions below, the common mode factor $|\psi_{\ell p}|^2$ multiplying the

factored optical contractions is included in Φ_G and $\mathcal{H}_A$. This is the convention used for the final optical factors in the Letter.

E1M1: optical chirality and the full six-factor rate

The T_0T_2 terms in the optical-chirality contraction are

$$|\psi|^2 \left(\mathbf{e}^{T_0} \cdot \bar{\mathbf{b}}^{T_2} + \mathbf{e}^{T_2} \cdot \bar{\mathbf{b}}^{T_0}\right) = \frac{\psi\psi_{tu}^* + \psi_{tu}\psi^*}{2k^2} = \frac{\mathrm{Re}(\psi_{tu}\psi^*)}{k^2}, \tag{75}$$

which is purely real. Consequently,

$$\mathrm{Im}\left(|\psi|^2 \mathbf{e} \cdot \bar{\mathbf{b}}\right)^{(2)} = \frac{1}{k^2}\,\mathrm{Im}\left(\psi_t\psi_u^*\right), \tag{76}$$

so the ℓ-odd optical chirality itself is purely longitudinal at this order.

The complete analysed Raman rate is not exhausted by this single contraction. Define

$$\mathcal{X} = \psi_t\psi_u^*, \qquad \mathcal{Y} = \psi_{tu}\psi^*. \tag{77}$$

The six E1M1 optical factors of Eq. (44), expanded through $O(\epsilon^2)$, are

$$\begin{aligned} |\psi|^2 F_1^{(2)} &= \frac{\mathcal{Y}^*}{2k^2}, & |\psi|^2 F_2^{(2)} &= \frac{\mathcal{X} + (\mathcal{Y} + \mathcal{Y}^*)/2}{k^2}, & |\psi|^2 F_3^{(2)} &= \frac{\mathcal{Y}^*}{2k^2}, \\ |\psi|^2 F_4^{(2)} &= -\frac{\mathcal{Y}^*}{2k^2}, & |\psi|^2 F_5^{(2)} &= 0, & |\psi|^2 F_6^{(2)} &= -\frac{\mathcal{Y}}{2k^2}. \end{aligned} \tag{78}$$

For non-resonant G, retaining the real part odd in ℓ gives

$$\mathrm{Re}\,\Phi_{G,\|,\ell\text{-odd}}^{(2)} = \frac{1}{k^2}\left[\mathrm{Im}(\mathcal{Y})\mathcal{G}_1 - \mathrm{Im}(\mathcal{X})\mathcal{G}_{\mathrm{ROA}}\right]. \tag{79}$$

Thus the T_0T_2 pair cancels from optical chirality but the other isotropic Raman contractions

leave a distinct T_2-activated $\mathcal{G}_1$ contribution.

E1E2 through $O(\epsilon^2)$

For a real parallel analyser the master contraction Eq. (53) can be written exactly in the $(\mathbf{t}, \mathbf{u}, \hat{\mathbf{z}})$ basis as

$$\mathcal{H}_{A,\text{in}} = e_z\psi\left(\partial_t(\bar{e}_u\bar{\psi}) + \partial_u(\bar{e}_t\bar{\psi})\right) - e_u\psi\left(\partial_t(\bar{e}_z\bar{\psi}) + \partial_z(\bar{e}_t\bar{\psi})\right), \tag{80}$$

$$\mathcal{H}_{A,\text{out}} = -ik'|\psi|^2\left(2e_t\bar{e}_u - e_u\bar{e}_t\right). \tag{81}$$

Through $O(\epsilon^2)$,

$$\mathcal{H}_{A,\text{in}}\big|_{O(\epsilon^2)} = \frac{i}{k}\psi_t\psi_u^* + \frac{i}{2k}\psi_{tu}\psi^*, \tag{82}$$

$$\mathcal{H}_{A,\text{out}}\big|_{O(\epsilon^2)} = -\frac{ik'}{2k^2}\left(2\psi\psi_{tu}^* - \psi_{tu}\psi^*\right). \tag{83}$$

All terms containing the co-polarised scalar $S2$ field vanish from this parallel chiral bracket at $O(\epsilon^2)$: they require a zeroth-order $\mathbf{u}$ component of the electric field, which does not exist. The surviving T_2 correction is therefore generated by the cross-polarised V_2 field.

The real part is

$$\boxed{\operatorname{Re}\mathcal{H}_{A,\parallel}^{(2)} = \frac{1}{k}\operatorname{Re}\left(i\psi_t\psi_u^*\right) - \frac{1}{2}\left(\frac{1}{k} + \frac{3k'}{k^2}\right)\operatorname{Im}\left(\psi_{tu}\psi^*\right).} \tag{84}$$

The first term is the $L_1\nabla_\perp T_0$ contribution. The second is the complete surviving T_2 correction for this channel. In particular, the incident-gradient term $T_2\,\partial_z T_0$ survives, while its naive $T_0\,\partial_z T_2$ partner is removed by the fifth-rank molecular and parallel-polarisation contraction; the outgoing-gradient E2 time ordering provides the remaining same-order term.

LG reduction and the W and U weights

At the waist,

$$\psi_{\ell p} = f_{\ell p}(r) e^{i\ell\phi}, \qquad f_{\ell p}(r) = f_{\rm LG}^{|\ell| p}(r,0) \in \mathbb{R},$$
$$g_{\ell p}(r) = \frac{f'_{\ell p}(r)}{f_{\ell p}(r)}. \tag{85}$$

With $\vartheta = \phi - \chi$,

$$\psi_t = \psi \left(g_{\ell p} \cos\vartheta - \frac{i\ell}{r} \sin\vartheta \right),$$
$$\psi_u = \psi \left(g_{\ell p} \sin\vartheta + \frac{i\ell}{r} \cos\vartheta \right), \tag{86}$$

and

$$\mathrm{Re}\left(i\psi_t \psi_u^* \right) = \frac{\ell f_{\ell p}^2}{r} g_{\ell p}, \tag{87}$$
$$\mathrm{Im}\left(\psi_{tu} \psi^* \right) = \frac{\ell f_{\ell p}^2}{r} \left(g_{\ell p} - \frac{1}{r} \right) \cos 2(\phi - \chi). \tag{88}$$

Define

$$W_{\ell p} = -\frac{1}{k^2} \mathrm{Im}\left(\psi_t \psi_u^* \right), \qquad U_{\ell p}^{(\chi)} = \frac{1}{k^2} \mathrm{Im}\left(\psi_{tu} \psi^* \right). \tag{89}$$

The waist-plane reduction above makes both factors explicitly odd under $\ell \to -\ell$. Away from the waist, $W_{\ell p}$ remains odd, but the wavefront-curvature phase generally adds an ℓ-even part to $U_{\ell p}^{(\chi)}$ as written; the explicit ℓ-odd projection must then be taken when constructing $\Delta I_\ell^{\parallel}$. Explicitly, at $z = 0$,

$$W_{\ell p}(r, 0) = f_{\ell p}^2(r) \frac{\ell}{k^2 r} g_{\ell p}(r), \tag{90}$$
$$U_{\ell p}^{(\chi)}(r, \phi, 0) = f_{\ell p}^2(r) \frac{\ell}{k^2 r} \left(g_{\ell p}(r) - \frac{1}{r} \right) \cos 2(\phi - \chi). \tag{91}$$

The U term is local, polarisation aligned, and has zero complete azimuthal average.

Equations (79) and (84) become

$$\operatorname{Re}\Phi^{(2)}_{G,\|,\ell\text{-odd}} = W_{\ell p}\mathcal{G}_{\mathrm{ROA}} + U^{(\chi)}_{\ell p}\mathcal{G}_1, \tag{92}$$

$$\operatorname{Re}\mathcal{H}^{(2)}_{A,\|,\ell\text{-odd}} = kW_{\ell p} - \frac{k+3k'}{2}U^{(\chi)}_{\ell p}. \tag{93}$$

The complete local topological-charge differential through $O(\epsilon^2)$ is therefore

$$\boxed{\Delta I^{\|}_{\ell} = \frac{NI_{\mathrm{inc}}k'^4}{120\pi^2\varepsilon_0^2}\left\{\frac{1}{c}\left[W_{\ell p}\mathcal{G}_{\mathrm{ROA}} + U^{(\chi)}_{\ell p}\mathcal{G}_1\right] + \left[kW_{\ell p} - \frac{k+3k'}{2}U^{(\chi)}_{\ell p}\right]\mathcal{A}_{\mathrm{ROA}}\right\}.} \tag{94}$$

This is the SI form of the principal result in the revised Letter. Complete azimuthal averaging removes $U^{(\chi)}_{\ell p}$ and recovers the rotationally symmetric $W_{\ell p}$-only result. For complete, uniformly weighted collection over the entire transverse plane, the remaining contribution also cancels. At the beam waist,

$$\begin{aligned}\int_0^{2\pi} U^{(\chi)}_{\ell p}(r,\phi,0)\,d\phi &= 0,\\ \int_0^{2\pi} d\phi\int_0^{\infty} r\,dr\,W_{\ell p}(r,0) &= \frac{2\pi\ell}{k^2}\int_0^{\infty} f_{\ell p}(r)f'_{\ell p}(r)\,dr\\ &= \frac{\pi\ell}{k^2}\left[|f_{\ell p}(r)|^2\right]_0^{\infty} = 0.\end{aligned} \tag{95}$$

Here $f_{\ell p}(0) = f_{\ell p}(\infty) = 0$ for $|\ell| > 0$. Thus complete uniform transverse-plane collection eliminates both the $U^{(\chi)}_{\ell p}$ and $W_{\ell p}$ contributions. Observation of a nonzero net topological-charge differential therefore requires finite-aperture, annular, confocal, spatially resolved, or otherwise nonuniform transverse collection.

Linearly polarised input with unresolved output

For unresolved output,

$$I_{\text{unres}} = I_s + I_p, \tag{96}$$

with $\mathbf{e}'_s = \hat{\mathbf{s}}$ and $\mathbf{e}'_p = \hat{\mathbf{p}}$. The full E1M1 contribution is obtained by summing Eq. (43) over these two states with the complete $\mathrm{T}_0 + \mathrm{L}_1 + \mathrm{T}_2$ fields.

For the E1E2 contribution, the complete local waist-plane result can be written compactly by defining

$$V_{\ell p}(r,\phi) = f_{\ell p}^2(r)\frac{\ell}{k^2 r}\left(g_{\ell p}(r) - \frac{1}{r}\right)\cos 2\phi. \tag{97}$$

The part of the $s + p$ optical factor that is real and odd in ℓ is

$$\begin{aligned}\mathrm{Re}\,\mathcal{H}^{(2)}_{A,\text{unres},\ell\text{-odd}} = kW_{\ell p}\cos 2\chi\,\sin^2\theta + \frac{k}{2}V_{\ell p}\sin^2\theta \\ + \left[k + 3k'\cos\theta - 3k\cos^2\theta\right]U^{(\chi)}_{\ell p}.\end{aligned} \tag{98}$$

The first term is the $\mathrm{T}_0 + \mathrm{L}_1$ result. The final two terms are the missing local T_2 contribution. Thus the complete local E1E2 response does *not* have a forward/backward null:

$$\begin{aligned}\theta = 0: &\qquad (3k' - 2k)U^{(\chi)}_{\ell p}, \\ \theta = \pi: &\qquad -(2k + 3k')U^{(\chi)}_{\ell p}.\end{aligned} \tag{99}$$

Both $U^{(\chi)}_{\ell p}$ and $V_{\ell p}$ vanish under complete azimuthal averaging. Hence

$$\overline{\mathrm{Re}\,\mathcal{H}^{(2)}_{A,\text{unres},\ell\text{-odd}}}^{\,\phi} = kW_{\ell p}\cos 2\chi\,\sin^2\theta, \tag{100}$$

and the azimuthally averaged E1E2 intensity differential is

$$\boxed{\overline{\Delta I}^{\mathrm{unres},\phi}_{\ell,\alpha A} = \frac{N I_{\mathrm{inc}} k'^4}{120\pi^2\varepsilon_0^2} W_{\ell p}\, k\mathcal{A}_{\mathrm{ROA}} \cos 2\chi\, \sin^2\theta.} \tag{101}$$

The familiar forward/backward suppression therefore applies to the azimuthally averaged channel, not to the complete pointwise second-order signal.

Scattered circular-polarisation detection

The far-field circular analyser states are

$$\mathbf{e}'_\tau = \frac{1}{\sqrt{2}}\left(\hat{\mathbf{s}} + i\tau\hat{\mathbf{p}}\right), \qquad \tau = \pm 1. \tag{102}$$

They satisfy

$$\begin{aligned} \mathbf{b}'_\tau &= \hat{\mathbf{k}}' \times \mathbf{e}'_\tau = -i\tau\mathbf{e}'_\tau, \\ \mathbf{c}'_\tau &= \mathbf{e}'_\tau \times \bar{\mathbf{e}}'_\tau = -i\tau\hat{\mathbf{k}}'. \end{aligned} \tag{103}$$

The scattered circular-polarisation differential and the vortex-specific double differential are

$$\begin{aligned} \Delta^{\ell}_{\mathrm{SCP}} &= I_{\tau=+}(\ell) - I_{\tau=-}(\ell), \\ \Delta_{\ell,\mathrm{SCP}} &= \Delta^{+\ell}_{\mathrm{SCP}} - \Delta^{-\ell}_{\mathrm{SCP}}. \end{aligned} \tag{104}$$

This channel is evaluated by substituting Eq. (102) directly into the full six-factor E1M1 rate, Eq. (43), and the full-gradient E1E2 master contraction, Eq. (53), expanding the resulting products, and retaining their total Lax order consistently through $O(\epsilon^2)$. This channel remains a useful experimental comparison but mixes the vortex-dependent field structure with the circular output analyser and is not the primary Letter observable.

Circularly polarised vortex input

For incident circular polarisation,

$$\mathbf{t}_\sigma = \frac{1}{\sqrt{2}}\left(\hat{\mathbf{x}} + i\sigma\hat{\mathbf{y}}\right), \qquad \alpha_\sigma = \frac{1}{\sqrt{2}}, \qquad \beta_\sigma = \frac{i\sigma}{\sqrt{2}}, \qquad \sigma = \pm 1. \tag{105}$$

The rotated transverse vector obeys

$$\mathbf{u}_\sigma = \hat{\mathbf{z}} \times \mathbf{t}_\sigma = -i\sigma\mathbf{t}_\sigma. \tag{106}$$

Because the L_1 and T_2 operators are linear in the seed transverse vector,

$$\boxed{\mathbf{b}_{\sigma,\ell p} = -i\sigma\mathbf{e}_{\sigma,\ell p}} \qquad \text{through } \mathrm{T}_0 + \mathrm{L}_1 + \mathrm{T}_2. \tag{107}$$

Thus the focused circular vortex remains a helicity eigenfield within this dual-symmetrized Lax expansion.

The longitudinal derivative factor is

$$\psi_{t_\sigma} = \frac{1}{\sqrt{2}}\left(\partial_x + i\sigma\partial_y\right)\psi. \tag{108}$$

At the waist,

$$\psi_{t_\sigma} = \frac{e^{i(\ell+\sigma)\phi}}{\sqrt{2}}\left(f'_{\ell p} - \frac{\sigma\ell}{r}f_{\ell p}\right). \tag{109}$$

Using Eq. (107),

$$\mathrm{Im}\left(|\psi_{\ell p}|^2\mathbf{e}_{\sigma,\ell p}\cdot\bar{\mathbf{b}}_{\sigma,\ell p}\right) = \sigma|\psi_{\ell p}|^2\left|\mathbf{e}_{\sigma,\ell p}\right|^2. \tag{110}$$

Expanding the electric-field norm through $O(\epsilon^2)$ gives

$$f_{\ell p}^2 \left|\mathbf{e}_{\sigma,\ell p}\right|^2 = f_{\ell p}^2 + \frac{f_{\ell p}^2}{2k^2}\left(g_{\ell p} - \frac{\sigma\ell}{r}\right)^2 + \frac{f_{\ell p}^2}{k^2}\left[\frac{f''_{\ell p}}{f_{\ell p}} + \frac{g_{\ell p}}{r} - \frac{\ell^2}{r^2}\right] + O(\epsilon^4 f_{\ell p}^2). \tag{111}$$

Therefore

$$\boxed{\frac{\mathrm{Im}\left(f_{\ell p}^2 \mathbf{e}_{\sigma,\ell p}\cdot\bar{\mathbf{b}}_{\sigma,\ell p}\right)}{f_{\ell p}^2} = \sigma\left\{1 + \frac{1}{2k^2}\left(g_{\ell p} - \frac{\sigma\ell}{r}\right)^2 + \frac{1}{k^2}\left[\frac{f''_{\ell p}}{f_{\ell p}} + \frac{g_{\ell p}}{r} - \frac{\ell^2}{r^2}\right]\right\} + O(\epsilon^4).} \tag{112}$$

The final bracket is the $\mathrm{T}_0\mathrm{T}_2$ correction. It is even in ℓ, whereas the vortex-odd part is

$$\left[\frac{\mathrm{Im}(f_{\ell p}^2 \mathbf{e}_{\sigma,\ell p}\cdot\bar{\mathbf{b}}_{\sigma,\ell p})}{f_{\ell p}^2}\right]_{\ell\text{-odd}} = -\frac{\ell}{k^2 r} g_{\ell p}. \tag{113}$$

Consequently, fixed-helicity topological-charge reversal removes both the ordinary spin term and the ℓ-even T_2 correction:

$$\Delta_\ell^\sigma = I^{\sigma,+\ell} - I^{\sigma,-\ell}, \tag{114}$$

$$\Delta\left[\mathrm{Im}(f_{\ell p}^2 \mathbf{e}\cdot\bar{\mathbf{b}})\right]_\ell^\sigma = -2f_{\ell p}^2 \frac{\ell}{k^2 r} g_{\ell p} = -2W_{\ell p}. \tag{115}$$

By contrast, incident-helicity reversal at fixed ℓ,

$$\Delta_\sigma^\ell = I^{+,\ell} - I^{-,\ell}, \tag{116}$$

retains the conventional circular-ROA background together with ℓ-even focusing corrections. The simultaneous reversal

$$\Delta_{\sigma\ell} = I^{+,+\ell} - I^{-,-\ell} \tag{117}$$

is a combined spin–vortex control rather than a pure vortex observable.

Equations (112)–(115) refer specifically to the E1M1 optical-chirality contraction. The complete polarisation-analysed circular-input Raman rate also contains the other E1M1 contractions and the E1E2 gradient terms. These are obtained by substituting $\alpha = 1/\sqrt{2}$, $\beta = i\sigma/\sqrt{2}$ into Eqs. (43) and (53), with $\mathbf{e}_{\ell p}$, $\mathbf{b}_{\ell p}$, and D_{ml} defined through T_2 above, and retaining the resulting contractions consistently through $O(\epsilon^2)$.

Summary of retained second-order terms

The second-order contributions retained in the topological-charge-dependent chiral signal can be summarized as

$$\begin{aligned} E1M1: &\quad \mathrm{L}_1\,\mathrm{L}_1 + \mathrm{T}_0\,\mathrm{T}_2, \\ E1E2: &\quad \mathrm{L}_1\,\nabla_\perp \mathrm{T}_0 + \mathrm{T}_2\,\partial_z \mathrm{T}_0 + \mathrm{T}_0\,\partial_z \mathrm{T}_2 + \text{outgoing-gradient } \mathrm{T}_0\,\mathrm{T}_2. \end{aligned} \tag{118}$$

All these terms have total beam order $O(\epsilon^2)$. The $\mathrm{T}_0\,\partial_z \mathrm{T}_2$ contribution is present in the general E1E2 expansion but cancels in the parallel-analysed linear-polarisation contraction used for the principal result. In the parallel linear channel, the $\mathrm{T}_0\mathrm{T}_2$ pair also cancels from the mode-weighted optical-chirality contraction $|\psi|^2\,\mathrm{Im}(\mathbf{e}\cdot\bar{\mathbf{b}})$, but the full E1M1 Raman average retains $U_{\ell p}^{(\chi)}\mathcal{G}_1$. The E1E2 molecular and polarisation contraction does not exhibit the corresponding $\mathrm{T}_0\mathrm{T}_2$ cancellation and retains the $U_{\ell p}^{(\chi)}$ correction in the complete local signal. Both $U_{\ell p}^{(\chi)}$ and the additional unresolved-output $V_{\ell p}$ harmonic vanish under complete azimuthal averaging.
